# Discovery of a Novel Chemo-Type for TAAR1 Agonism via Molecular Modeling

**DOI:** 10.3390/molecules29081739

**Published:** 2024-04-11

**Authors:** Giancarlo Grossi, Naomi Scarano, Francesca Musumeci, Michele Tonelli, Evgeny Kanov, Anna Carbone, Paola Fossa, Raul R. Gainetdinov, Elena Cichero, Silvia Schenone

**Affiliations:** 1 Department of Pharmacy, Section of Medicinal Chemistry, School of Medical and Pharmaceutical Sciences, University of Genoa, Viale Benedetto XV, 3, 16132 Genoa, Italy; giancarlo.grossi@unige.it (G.G.); naomi.scarano@edu.unige.it (N.S.); francesca.musumeci@unige.it (F.M.); michele.tonelli@unige.it (M.T.); anna.carbone1@unige.it (A.C.); paola.fossa@unige.it (P.F.); silvia.schenone@unige.it (S.S.); 2 Institute of Translational Biomedicine, St. Petersburg State University, 199034 St. Petersburg, Russiagainetdinov.raul@gmail.com (R.R.G.); 3 St. Petersburg University Hospital, St. Petersburg State University, 199034 St. Petersburg, Russia

**Keywords:** AlphaFold, molecular docking, TAAR1, agonist, trace amine receptor

## Abstract

The search for novel effective TAAR1 ligands continues to draw great attention due to the wide range of pharmacological applications related to TAAR1 targeting. Herein, molecular docking studies of known TAAR1 ligands, characterized by an oxazoline core, have been performed in order to identify novel promising chemo-types for the discovery of more active TAAR1 agonists. In particular, the oxazoline-based compound **S18616** has been taken as a reference compound for the computational study, leading to the development of quite flat and conformationally locked ligands. The choice of a “Y-shape” conformation was suggested for the design of TAAR1 ligands, interacting with the protein cavity delimited by ASP103 and aromatic residues such as PHE186, PHE195, PHE268, and PHE267. The obtained results allowed us to preliminary in silico screen an in-house series of pyrimidinone-benzimidazoles (**1a**–**10a**) as a novel scaffold to target TAAR1. Combined ligand-based (LBCM) and structure based (SBCM) computational methods suggested the biological evaluation of compounds **1a**–**10a**, leading to the identification of derivatives **1a**–**3a** (hTAAR1 EC_50_ = 526.3–657.4 nM) as promising novel TAAR1 agonists.

## 1. Introduction

Trace amines (TAs) are a group of endogenous chemical messengers closely related to the biogenic amine neurotransmitters, i.e., dopamine (DA), serotonin (5-HT), and norepinephrine (NE). Among them, β-phenylethylamine (β-PEA), *p*-tyramine, tryptamine, *p*-octopamine, and others are found in both invertebrate and vertebrate species [1,2,3]. The Trace Amine-Associated Receptor (TAAR) family belongs to the class A G-protein coupled receptors (GPCRs) and consists of six functional members in humans (TAAR1, 2, 5, 6, 8, and 9), with TAAR1 being the most investigated. TAARs are widely expressed at low levels in the brain and periphery [4]. In the central nervous system (CNS), it was originally believed that the TAAR2–TAAR9 subtypes were expressed primarily in the olfactory system, and indeed a role in olfaction has been identified for most of them [5,6,7,8]. However, recent data indicated they are also expressed in limbic brain areas receiving olfactory input and involved in the regulation of emotional behaviors and adult neurogenesis [9,10,11]. By contrast, TAAR1 has no known role in olfaction, and in rodents is highly expressed in the ventral tegmental area and dorsal raphe nuclei [12,13,14], brain regions involved in dopaminergic and serotonergic signaling, respectively. Recently, an association between mutations in the TAAR1 gene and schizophrenia was reported [15,16], suggesting the implication of TAAR1 in the pathways driving schizophrenia [17].

In this scenario, targeting TAAR1 could lead to novel approaches for the treatment of several disorders [18,19,20] including not only schizophrenia but also depression, attention deficit hyperactivity disorder, addiction [21], and metabolic diseases by means of agonist compounds, and Parkinson’s disease by antagonists [22,23,24,25,26,27].

During recent years, the development of TAAR1 ligands has arisen from the main chemical scaffold of the endogenous TAs, leading to the identification of several analogues often endowed with a more promising mouse TAAR1 (mTAAR1) affinity, rather than towards the human ortholog (hTAAR1) [3,28]. This preliminary information highlighted the species-specificity issues complicating virtual screening campaigns and the rational design processes aimed towards novel hTAAR1 effective ligands [29,30,31,32].

In this context, we recently explored via molecular modeling studies, followed by chemical synthesis and biological evaluation, different series of more selective m/hTAAR1 ligands, obtaining in silico the main features for selective compounds [33,34,35].

Hoffmann-La Roche was an early leader in TAAR1 ligand discovery, relying on high-throughput screening (HTS) and repositioning approaches. Indeed, the discovery of novel TAAR1 agonists exploited libraries of known dopaminergic, serotonergic, and adrenergic drugs [36,37,38,39,40] and a number of promising TAAR1 agonists has been discovered [41,42]. 

**Figure 1:**
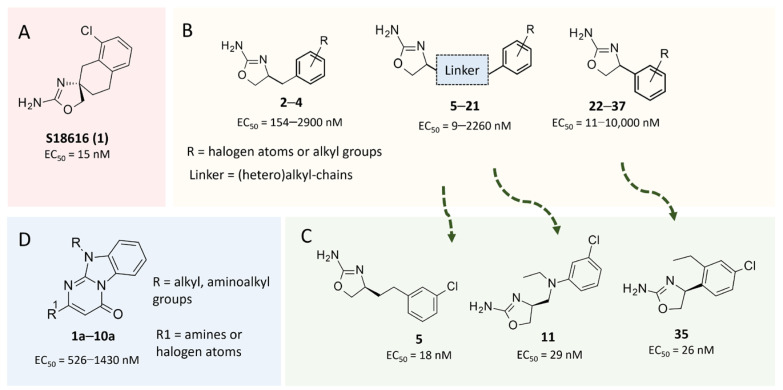
Structural variations performed at the **S18616 1** (**A**) [43] compound as TAAR1 prototype towards further oxazoline-based agonists **2**–**37** (**B**) [38]. The promising compounds **5**, **11,** and **35** have been pointed out (**C**). The chemical structure of the herein proposed TAAR1 agonists **1a**–**10a** is shown (**D**). The corresponding hTAAR1 EC_50_ values are reported.

Among them, compound **S18616 1** (Figure 1A) was reported in the literature as a potent alpha_2_-adrenoreceptor (α_2_-ADR) agonist, being then also evaluated as a TAAR1 agonist [43]. 

To pursue more selective TAAR1 or α_2_-ADR ligands, structural simplification of the main **S18616 1** tricyclic ring has been afforded, leading to different series of more selective TAAR1 ligands [36,37] such as oxazoline derivatives (**2**–**37**) [38]. As shown in Figure 1B, these compounds can be mainly divided into benzyl-based ones (**2**–**4**), phenyl (hetero)alkyl-containing compounds (**5**–**21**), and phenyl-based derivatives (**22**–**37**).

The most promising identified TAAR1 agonists exhibit: (i) a flexible main spacer tethering the oxazoline core and the terminal aromatic ring (see **5** as prototype of the alkyl-phenyl-based analogues; Figure 1C), (ii) a folded/branched conformation due to a heteroalkyl spacer in the molecule (see **11** as prototype of the heteroaryl-containing analogues; Figure 1C), (iii) rigid positioning due to a phenyl ring directly connected to the main oxazoline core (see **35** as prototype of the phenyl-based analogues; Figure 1C). 

These data provide useful guidelines for the design of novel putative TAAR1 agonists. Molecular modeling approaches, such as ligand- and structure-based computational methods, represent widely exploited complemental tools to guide the discovery of novel bioactive molecules [44,45,46,47,48,49,50]. While a number of homology models of m/hTAAR1 have been already reported by us or described in the literature [51,52,53,54], the human TAAR1 model was recently determined thanks to the AlphaFold protein structure database (AF-Q96RJ0-F1) [55,56]. Notably, AlphaFold is used to predict the structures of almost all of the proteins in the human proteome giving high-confidence predicted structures towards new avenues of investigation from a structural perspective [57]. However, homology modeling strategies and deep learning AlphaFold could be complementary, running in different ways and giving different applicability based on the protein of interest [58].

This information prompted us to explore in silico via molecular docking studies the aforementioned hTAAR1 agonist prototype **S18616 1** and the expanded series of oxazoline derivatives **1**–**37** (see the chemical structures in Table S1). The results were expected to provide more information on the putative binding mode of flexible or not TAAR1 ligands at the protein binding cavity and to pave the way for the following in silico evaluation of novel TAAR1 agonists. Indeed, the in-house small library of compounds **1a**–**10a** (Figure 1D), previously reported as human platelet aggregation inhibitors [59], has been herein studied as putative TAAR1 ligands (see the chemical structures in Table S2). The final aims of this study focused on the discovery of novel chemo-types endowed with TAAR1 agonism ability.

Compounds **1a**–**10a** are characterized by a flat and mainly aromatic tricyclic ring (Figure 1D) mimicking the main core of the reference compound **S18616 1** (Figure 1A). In particular, the **1a**–**10a** pyrimidinone ring is expected to mimic the oxazoline ring of the TAAR1 agonists **1**–**37**. In addition, most of the compounds **1a**–**10a** maintained a key basic moiety in R^1^, to simulate the compound **S18616 1** primary amine group.

Herein, in silico investigation of **1a**–**10a** has been performed combining ligand-based computational methods (LBCMs) (FLAP2.2.1. software) [60,61] and structure-based ones (SBCMs), such as molecular docking calculations by means of the AlphaFold hTAAR1 model. Using the results of the aforementioned **1**–**37** docking mode as a filtering tool and combining the results of the two computational strategies led to the biological evaluation of ten compounds as putative hTAAR1 ligands. 

The subsequent in vitro evaluation confirmed the hTAAR1 agonism ability featured by six out of the ten proposed compounds (**1a**–**6a**), showing for the **1a** derivative (hTAAR1 EC_50_ = 526.3 nM) the lowest EC_50_ value among the in silico evaluated analogues (hTAAR1 EC_50_ = 526.3–1.43 μM). Finally, the whole study has been completed by in silico prediction of descriptors related to absorption, distribution, metabolism, and excretion properties (ADME) of **1a**–**6a**, in order to prioritize the most promising newly discovered TAAR1 ligands. The obtained results in this study allowed us to validate the computational protocol towards the identification of a novel scaffold for the design of novel TAAR1 agonists. The biological data in tandem with the reported ADME prediction have supported the proposed scaffold as being worthy of further optimization.

## 2. Results

During the last years, a number of structure-based studies and ligand-based approaches have been attempted to rationalize the structure–activity relationship (SAR) of different series of TAAR1 agonists, such as imidazole- and imidazoline-based compounds [62], as well as a limited number of oxazolines endowed with hTAAR1 agonism ability [35]. Most of the previously investigated derivatives shared the key structural features required for a TAAR1-targeting activity, as a basic core forming a key salt-bridge with a conserved hTAAR1 aspartic acid (ASP103) and an aromatic moiety forming π–π stacking and van der Waals interactions with the surrounding residues.

Very recently, we pointed out the relevant role played by two aromatic cores endowed with H-bonding features within the TAAR1 agonist, to be folded as a “Y-shape” conformation [63], as it is in the case of other GPCR ligands [64,65].

Based on the hTAAR1 AlphaFold protein model, herein we preliminary explored the effectiveness of structural variations afforded to the main scaffold of the conformationally locked hTAAR1 agonist **S18616 1**, via molecular docking of the analogues **2**–**37** (see the chemical structures in Table S1). Details of the calculated scoring functions are listed in Table S3. Most of the oxazolines **1**–**37** featured S values spanning from −4.6935 Kcal/mol, such as **15** (hTAAR1 EC_50_ = 1540 nM), to −7.1321 Kcal/mol (**21**; hTAAR1 EC_50_ = 9 nM), with the reference compound **S18616** being endowed with S = −6.8545 Kcal/mol.

As shown in Table 1, a perspective of compound **1** and of compounds **2**–**21** [38,43], as flexible oxazoline-based TAAR1 agonists, is reported. The predicted ΔG value of each best-scored protein–agonist complex has been listed, based on the performed molecular docking calculations. 

Regarding the phenyl-based oxazoline compounds **22**–**37** [38,43], the predicted ΔG value of each best-scored protein–agonist complex is reported in Table 2.

The obtained results are expected to (i) reveal more information about the protein–agonist interactions in presence of differently flexible ligands as those herein considered, and (ii) point out useful guidelines to identify novel promising chemo-types for the development of novel TAAR1 agonists. Indeed, compounds **1a**–**10a** have been evaluated in silico and described as follows, thanks to LBCM (FLAP software) [60,61] in tandem with molecular docking studies, as a structure-based tool. Based on the combined LB- and SBCM results, ten compounds have been assayed as putative TAAR1 agonists. Finally, the final biological evaluation of compounds **1a**–**10a** allowed us to confirm the reliability of the computational studies.

### 2.1. Molecular Docking Studies of Oxazoline-Based TAAR1 Agonists

**Figure 2:**
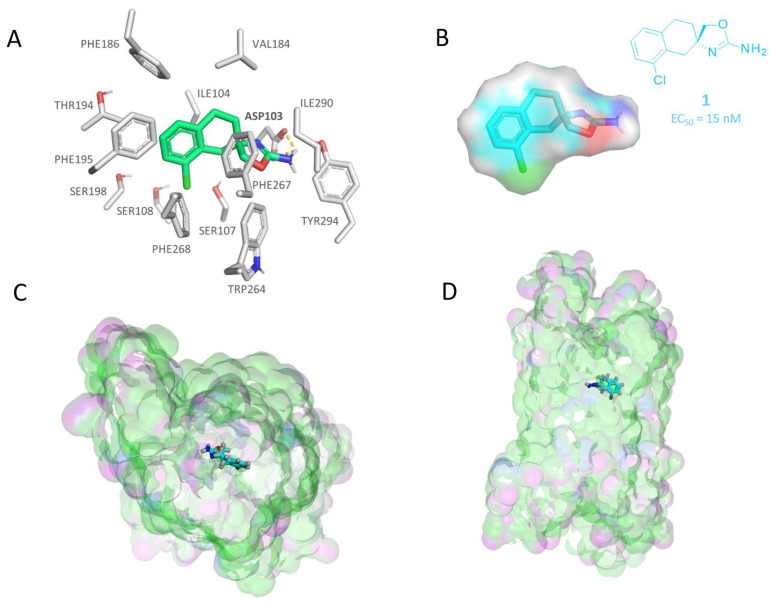
Molecular docking pose of compound **S18616 1** [43] (C atom; light green) within the hTAAR1 binding site (**A**). The most important residues are shown and labeled. H-bonds are highlighted as dashed yellow lines. The TAAR1 agonist chemical structure and the ligand hydrophobicity/electrostatic properties are also reported (hydrophobic and mild polar areas are shown in green and cyan, respectively; electron-rich and -poor groups are highlighted in red and blue). (**B**). The top view and side view of the receptor in the presence of **S18616 1** are shown in (**C**,**D**), respectively. The H-bonding and hydrophobic areas of the protein are colored magenta and green. All the surface representations have been produced via MOE2019.01 software [67].

According to our molecular docking calculations, the compound **1** (**S18616**, EC_50_ = 15 nM) docking pose revealed an H-bond between the amino group of the ligand and the side chain of residue ASP103 (Figure 2A). This is probably an interaction key for bioactivity, as was also hypothesized for other TAAR1-targeting molecules [66].

Several other non-polar interactions were found between the aromatic six-membered ring of the molecule and residues PHE186, PHE195, and PHE268 (staggered π-stacking interaction). In addition, the cyclohexyl group was engaged in Van der Waals contacts with VAL184, ILE 104, PHE267, and ILE290. A perspective of the ligand and receptor hydrophobic/polar properties is reported in Figure 2B and Figure 2C,D, respectively.

Herein, compound **1** (**S18616**) was taken as a reference compound for a three-dimensional SAR analysis involving the other analogues **2**–**37**, based on compound **1**’s high potency as TAAR1 agonist and its conformationally locked binding pose.

The TAAR1 ligands **2**–**37** were derived by structural simplification of the previous derivative **1** opening the tricyclic ring and obtaining oxazoline-based phenyl-, benzyl-, phenyl (amino)alkyl-containing derivatives. 

The benzyl-based ones (**2**–**4**; hTAAR1 EC_50_ = 154–2900 nM) have been less explored due to their lower potency trend compared to **1**, whereas a number of phenyl (hetero)alkyl-containing compounds (**5**–**21**; EC_50_ = 9–2260 nM) and phenyl-based compounds (**22**–**37**; hTAAR1 EC_50_ = 11–10,000 nM) exhibited promising EC_50_ values.

Within the benzyl-based compound series, the opening of the central cycle of the reference **1** in tandem or not with the elimination of the chlorine atom on the phenyl ring was disadvantageous to the TAAR1 agonism ability, as featured by **3**–**4** (hTAAR1 EC_50_ = 330–2900 nM) and **2** (hTAAR1 EC_50_ = 154 nM), respectively. In addition, modifying the S enantiomer to the *R* one impaired the compound potency values, as confirmed by the two enantiomer compounds **3** (*S*) and **4** (*R*).

The introduction of a longer spacer tethering the main amino-oxazoline ring and the terminal phenyl group led to the effective phenylethyl- and propylethyl-containing analogues **5** (hTAAR1 EC_50_ = 18 nM), **6** (hTAAR1 EC_50_ = 27 nM), and **19** (hTAAR1 EC_50_ = 27 nM), respectively. This modification was favorable for activity, leading to a decrease in EC_50_ values of around one order of magnitude with respect to compounds **2** and **3**, being quite comparable with the prototype **1** (hTAAR1 EC_50_ = 15 nM). Moreover, it should be noticed that compounds **5** and **6** differ from each other by a chlorine atom on the phenyl ring (compound **5**), which seems to be further advantageous for activity.

**Figure 3:**
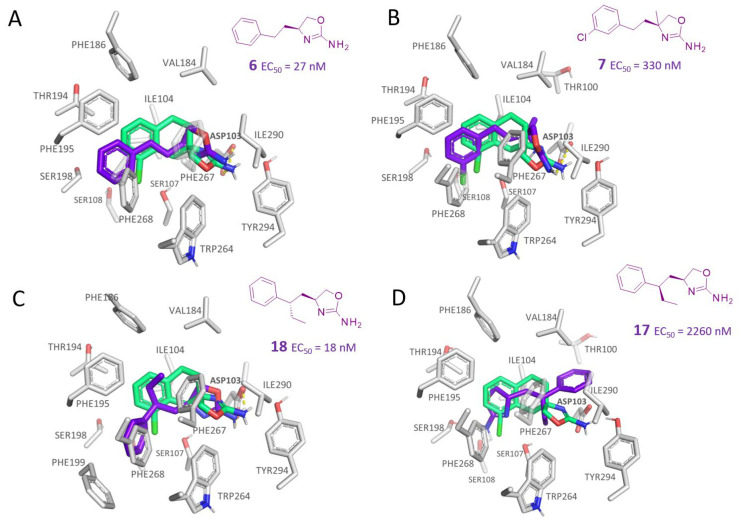
Molecular docking pose of compound **6** (C atom; violet) (**A**), **7** (C atom; violet) (**B**), **18** (C atom; violet) (**C**), and **17** (C atom; violet) (**D**) within the hTAAR1 binding site. The docking pose of the reference compound **S18616 1** [43] is reported (C atom; light green). The most important residues are shown and labeled. H-bonds are highlighted as dashed yellow lines. The chemical structures of the TAAR1 agonists **6**, **7** [38] and **17**, **18** [38] are reported.

As shown in Figure 3A, the best-scored docking pose of compound **6** (hTAAR1 EC_50_ = 27 nM) highlights a folded ligand conformation that allowed the compound to display the pivotal H-bond with the key residue ASP103, in tandem with additional π-π stacking with the surrounding aromatic residues. Indeed, a favorable interaction between the phenyl ring and PHE268 (T-shaped π-interaction) was highlighted, together with the insertion of the phenyl in a sub-pocket defined by SER198, SER108, SER 197, and PHE268. On the other hand, no hydrophobic contacts involving PHE186 were present.

The introduction of additional alkyl groups at the stereo-center or involving the ethyl-chain spacer was not advantageous, as shown by compounds **7** (EC_50_ = 330 nM) and **15**–**17** (hTAAR1 EC_50_ = 730–2260 nM), except for **18** (hTAAR1 EC_50_ = 18 nM). The lower activity of oxazoline **7** compared to the reference compound **1** was probably due to an unfavorable hindrance between the introduced group and the surrounding residues (VAL184, ILE104, and the key residue ASP103), as depicted in Figure 3B. This event moved the compound quite far from the ASP103 residue, making the corresponding H-bond weaker than that featured by the analogue **6**.

Conversely, compound **18** proved to better mimic the docking positioning observed for **1**, moving the primary amino group near ASP103 (see Figure 3C). 

The choice of the (*S*)-ethyl substituent branching the main ethyl spacer allowed simulation of the required hydrophobic features of the **S18616 1** cyclohexyl group, while the terminal phenyl ring was projected towards the aromatic residue PHE268. Accordingly, the corresponding (*R*)-ethyl substituted analogue **17** (hTAAR1 EC_50_ = 2260 nM) was differently folded (Figure 3D), exhibiting higher EC_50_ values than **18** (hTAAR1 EC_50_ = 18 nM).

**Figure 4:**
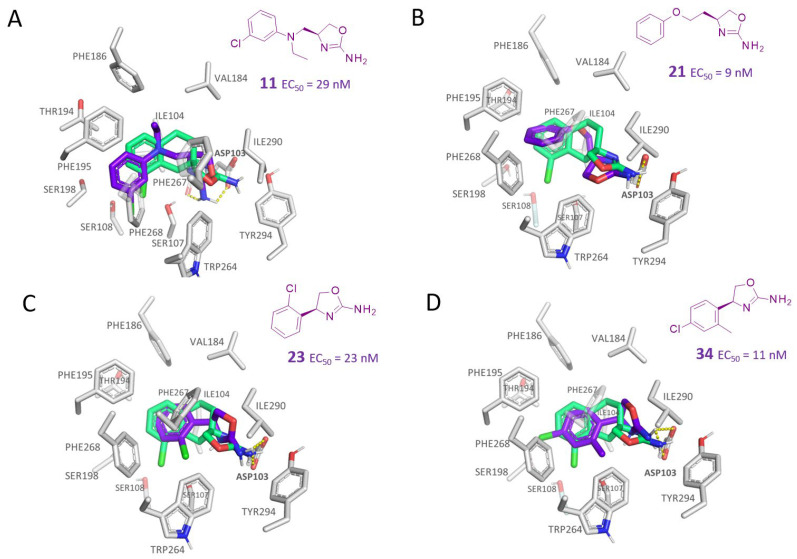
Molecular docking pose of compound **11** (C atom; violet) (**A**), **21** (C atom; violet) (**B**), **23** (C atom; violet) (**C**), and **34** (C atom; violet) (**D**) within the hTAAR1 binding site. The docking pose of the reference compound **S18616 1** [43] is reported (C atom; light green). The most important residues are shown and labeled. H-bonds are highlighted as dashed yellow lines. The chemical structures of the TAAR1 agonists **11, 21** [38] and **23**, **34** [38] are reported.

As regards the phenyl heteroalkyl-containing compounds (**8–14, 20**–**21**; hTAAR1 EC_50_ = 9–580 nM), the introduction of H-bond acceptor moieties is preferred to that of H-bond donor ones, rendering the presence of tertiary amino groups and phenoxy moieties encouraged. Accordingly, compound **9** exhibiting a secondary amino group tethered to the main oxazoline core displayed lower EC_50_ values (hTAAR1 EC_50_ = 580 nM) than the congeners **10–11** (hTAAR1 EC_50_ = 27 nM), featuring a tertiary amino-containing substituent. The corresponding compound **11** docking pose revealed that the overall pattern of interactions with respect to the reference compound **S18616 1** was quite well maintained (Figure 4A). 

The H-bond with ASP103 is present and involved both the side chain and the oxygen backbone of the residue. It is possible to notice that the chloro-phenyl moiety exhibited a small displacement with respect to compound **1**, but with the chlorine atom pointing in the same direction. The presence of the ethyl substituent on the linker amine partially reproduced the hindrance and hydrophobicity of the junction between the aromatic and central cycle of the reference compound **S18616**.

According to these data, it is possible to hypothesize that a certain hydrophobicity in this area may be of relevance in ligand affinity, leading the ligand to establish better contacts with the surrounding residues (e.g., PHE195, PHE186, VAL184, ILE104, PHE267), or at least to avoid contacts between polar and non-polar groups. Notably, increasing the hindrance of the aliphatic substituent to iPr dimension as shown by **13** (hTAAR1 EC_50_ = 140 nM) resulted in decreased activity, possibly because of steric clashes with the mentioned residues. The presence of the chlorine atom, although not fundamental, was advantageous for activity [compare **11** (hTAAR1 EC_50_ = 29 nM) and **12** (hTAAR1 EC_50_ = 59 nM)], as it can insert in a small volume delimited by residues SER107, SER108, and PHE268. 

The presence of the phenoxy moiety combined with the overall three-atom spacer allowed compound **21** (hTAAR1 EC_50_ = 9 nM) to exhibit the pivotal folded conformation guiding the proper positioning of the ligand primary amino group near ASP103, as described for all the previous effective TAAR1 agonists (Figure 4B). In addition, stabilizing interactions were observed between the phenyl-ring of compound **21** and the surrounding aromatic residues (PHE195, PHE268, PHE267). 

The elimination of the linker, leading to a direct bond between the two cyclic systems, resulted in acceptable activity as shown by a consistent number of phenyl-based compounds (**22**–**37**; hTAAR1 EC_50_ = 11-10.000 nM), as reported for **22**–**24**, **26**, **30**, **33**–**37** (EC_50_ = 21–67 nM). The docking pose of compound **23** (hTAAR1 EC_50_ = 23 nM), taken as representative of these effective TAAR1 agonists, revealed a similar binding mode with respect to the reference compound **S18616**. As shown in Figure 4C, the phenyl ring is oriented similarly, and the H-bond with ASP103 is maintained. Small differences are highlighted, such as a variation in the chlorine orientation, and a different conformation of the oxazoline ring, lacking contacts with residue TRP264.

*Meta*- (**24**; hTAAR1 EC_50_ = 21 nM) and *para*- (**25**, hTAAR1 EC_50_ = 143 nM) chloro substitution were explored in addition to the previously cited *ortho*- analogue **23**. While the *meta*-substitution was well tolerated, the *para*-substitution led to a considerable increase in EC_50_. Then, *para*-bromo substitution (**27**; hTAAR1 EC_50_ = 150 nM) leads to a comparable activity with the previous compound **25**. Notably, when the *para*-Br substitution is combined with the stereocenter inversion to *R* configuration, a strong loss in activity occurred (**28**; hTAAR1 EC_50_ = 10,000 nM). Interestingly, dichloro substitution at the *meta*- and *para*- positions (**26**; hTAAR1 EC_50_ = 31 nM) led to a good activity. Compound **32** (hTAAR1 EC_50_ = 490 nM), with a fluorine in *meta*- position, exhibited a strong decrease in activity compared to the *meta*-chloro-containing analogue **24** (hTAAR1 EC_50_ = 21 nM). However, if this modification is accompanied by the presence of a methyl substituent in *ortho*- position, the activity is comparable to reference compound **1** (**37**; hTAAR1 EC_50_ = 17 nM). 

Accordingly, further to the halogen atom, the additional introduction of electron-donor substituents endowed with hydrophobic properties, such as aliphatic chains, led to improved TAAR1 agonists, as reported for **34**–**36** (hTAAR1 EC_50_ = 11–26 nM). Based on the molecular docking pose of the very potent compound **34** (hTAAR1 EC_50_ = 11 nM), the oxazoline is shifted towards residue VAL184, establishing good non-polar interactions between this residue and ILE290 (Figure 4D). The chlorine atom is inserted in a sub-pocket delimited by residues THR196, SER198, and PHE195. 

The presence of an ethyl- or cyclopropyl- group in place of the methyl in ortho also maintained an excellent activity, as reported by compounds **35** (hTAAR1 EC_50_ = 26 nM) and **36** (hTAAR1 EC_50_ = 12 nM). Bulkier substituents such as the diaryl moiety were not tolerated, as described for compound **31** (hTAAR1 EC_50_ = 2670 nM). Finally, the only insertion of the *ortho*-methyl substitution led to quite effective EC_50_ values [see **33** (hTAAR1 EC_50_ = 67 nM)]. 

### 2.2. In Silico Evaluation of Pyrimidinone-Benzimidazole-Based Compounds as TAAR1 Agonists

**Figure 5:**
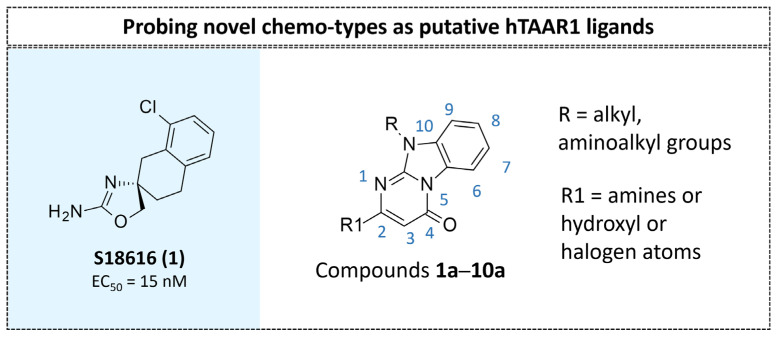
Schematic comparison of the herein investigated series of compounds **1a**–**10a** [59], with respect to the hTAAR1 prototype **S18616 1 [43]**.

According to the aforementioned **1**–**37** molecular docking studies, H-bonding to ASP103 and featuring π–π stacking with aromatic residues such as PHE186, PHE195, TRP264, and PHE268 was advantageous for TAAR1 agonism. This pharmacophore feature was achieved by the most effective compounds of the **1**–**37** series, exhibiting a branched structure endowed with H-bonding and aromatic features. As a result, the TAAR1 agonist “Y-shape” conformation was encouraged. On this basis, we focused on a small library of pyrimidinone-benzimidazole-based compounds **1a**–**10a**, previously reported by us as human platelet aggregation inhibitors (see the chemical structures in Table S2) [59], to be evaluated in silico as putative TAAR1 ligands, prior to their biological evaluation. As shown in Figure 5, all compounds are characterized by a flat and mainly aromatic tricyclic ring mimicking the main core of the reference compound **S18616 1**.

While the pyrimidinone ring A featured comparable electrostatic properties and electron-rich atoms to the corresponding **1** oxazoline ring (ring A), the terminal six-membered ring of the benzimidazole portion guaranteed the proper aromatic moieties displayed by the prototype ring C. On the other hand, the central ring B of the **1a**–**10a** series was endowed with additional nitrogen atoms if compared to the **S18616 1** ring B. The previously reported oxazoline series **2**–**37** pointed out the effectiveness of proper heteroatoms within the main spacer tethering the two terminal A and C rings, therefore supporting this structural variation.

As shown in Figure 5, most of the compounds maintained a key basic moiety in R^1^, in order to simulate the compound **S18616 1** primary amine group, while only three compounds bearing a halogen atom (**7a**, **10a**) or a hydroxyl group (**9a**) in R^1^ have been taken into account.

Finally, the steric hindrance displayed by **1a**–**10a** should be in good agreement with that of **1**, even if the molecule volume and shape are different. Based on the above, we assessed in silico the putative TAAR1 agonism ability of the pyrimidinone-benzimidazole scaffold, using ligand- and structure-based virtual screening methods. 

Initially, we proceeded with a LBCM approach, using compound **S18616 1** as reference compound. The results of the LBCM were ordered according to the Glob-Prod parameter (see experimental section), being the corresponding score values reported in Table S4. As control compounds, the TAAR1 agonists **2**–**37** have been also evaluated via LBCM. As shown in Table S5, the benzyl-based ones (**2**–**4**; EC_50_ = 154–2900 nM) have been predicted as very promising (Glob-Prod = 0.5961–0.6494), due to the similar volume and dimension compared to **S18616**; most of the phenyl (hetero)alkyl-containing compounds (**6**, **7**, **9**, **13**–**15**, **17**–**21**; EC_50_ = 9–2260 nM) were shown as effective, featuring Glob-Prod values spanning from 0.4090 to 0.5047. The phenyl-based compounds (**22**–**37**; EC_50_ = 11–10,000 nM) have been predicted as the less comparable oxazolines with respect to the template **S18616**, being in any case endowed with hTAAR1 agonism ability. In this case, the corresponding Glob-Prod values spanned from 0.3051 to 0.4076. By a perspective of the activity values featured by all the **S18616** analogues **1**–**37**, a Glob-Prod value of 0.3000 has been herein considered as acceptable for the ligand-based evaluation of further hTAAR1 agonists. 

As regard the pyrimidinone-benzimidazoles **1a**–**10a**, three derivatives (**1a**–**3a**) were endowed with Glob-Prod values higher than 0.3000, while the analogues **4a**–**6a** spanned from 0.2700 to 0.3000. Compounds **7a**–**10a** displayed the lowest values of Glob-Prod in this series of compounds (Glob-Prod = 0.1051–0.2692). Then, we proceed with a 3D-analysis of the results via visual comparison of the pyrimidinone-benzimidazole candidates’ molecular interaction fields (MIFs) with respect to those of the template **S18616 1**, following the score order. The following MIF probes have been taken into account: (i) DRY to evaluate the role played by hydrophobic features, (ii) N1 to assess favorable interaction with the H-bond donor (HBD) probe, (iii) O to assess favorable interaction with the H-bond acceptor (HBA) probe.

**Figure 6:**
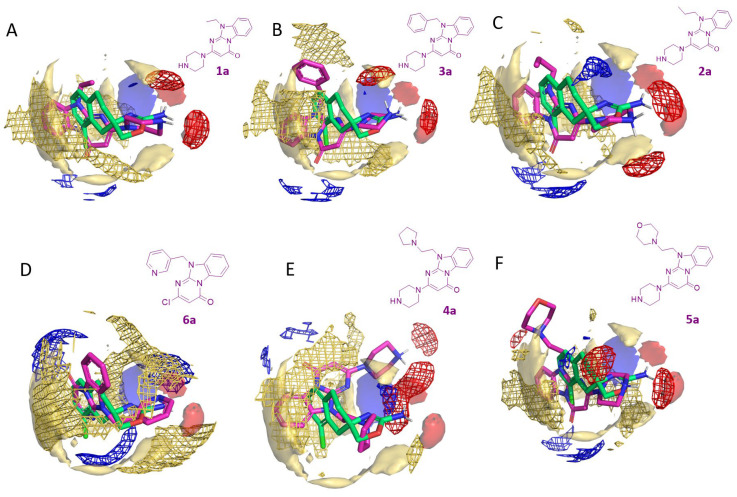
MIF Comparison of the template **S18686 1 [43]** (C atom; green) MIFs (solid) and those (wireframes) of the candidates [59] (C atom; magenta). Blue: N1 MIF, red: O MIF, yellow: DRY MIF. The analysis is reported for compounds **1a** (**A**), **3a** (**B**), **2a** (**C**), **6a** (**D**), **4a** (**E**), and **5a** (**F**) based on the calculated Glob-Prod ranking (**1a**, Glob-Prod = 0.3499; **3a**, Glob-Prod = 0.3167; **2a**, Glob-Prod = 0.3154; **6a**, Glob-Prod = 0.2962; **4a**, Glob-Prod = 0.2841; **5a**, Glob-Prod = 0.2838).

As regards the top-scored pyrimidinone benzimidazoles **1a**–**3a**, compound **1a** (Glob-Prod = 0.3499) displayed the highest Glob-Prod value (see Figure 6A). Good compatibility with the template **S18616 1** was observed for the three analyzed interactions (DRY, N1, and O MIFs, shown in yellow, blue, and red, respectively). 

The O MIF (red) of **1a** is well matching the template one in the area of the piperazine-oxazoline moieties, as the protonated piperazine ring of this compound mimics the amino-group of the amino-oxazoline of **S18686**. A small common area is also present for N1 MIF (blue MIF), as the oxazoline nitrogen displayed a similar interaction field as the piperazine tertiary amine of the candidate. DRY MIFs (yellow) largely overlap both on the front and back faces of the compounds, highlighting common interaction areas given by the aromatic/cyclic moieties and their hydrophobic substituents. 

Then, compound **3a** (Glob-Prod = 0.3167) exhibited overall good compatibility with the template, especially for the hydrophobic MIF (see Figure 6B). Again, the O MIF superposition only involved one of the two volumes shown by the template, as only one HBD of the candidate is coherently oriented with the **S18686** amine group. N1 MIF overlaps are negligible. Compound **2a** (Glob-Prod = 0.3154) exhibited similar behavior, with only one O MIF superimposed on the template one (see Figure 6C). In this case, the N1 MIF of the candidate was partially superimposed on the template one, as the piperazine tertiary amine (**2a**) mimics the oxazoline basic nitrogen (**S18686**). The DRY MIFs exhibit good superimposition on the two faces of the compounds. 

The following set of best-scored compounds included the analogues **4a**–**6a** (Glob-Prod = 0.2700 to 0.3000), which were predicted as the most promising of the last ones **7a**–**10a** (Glob-Prod = 0.1051–0.2692).

According to the Glob-Prod ranking, the pyrimidinone-benzimidazole **6a** (Glob-Prod = 0.2962) exhibited modest compatibility with the template, especially in terms of HBD and HBA interactions, while hydrophobic fields maintained a certain coherence with the template (see Figure 6D). 

Compound **4a** (Glob-Prod = 0.2841) displayed a small superposition between the O MIFs, in particular due to a similar orientation of the amino moiety of **S18686**, and the protonated pyrrolidine of the aforementioned **4a** (see Figure 6E). An overlap between N1 MIF was also present: the involved moieties were the oxazoline basic nitrogen and the tertiary amine of the piperazine ring of **4a**. However, such overlap derived from different areas of the molecules, and should not be regarded as a signal of ligand similarity. The overlap between DRY MIFs is strongly decreased, possibly due to the altered positioning of the tricyclic plane of **4a**. The analogue **5a** (Glob-Prod = 0.2838) displayed O MIFs superposition due to consistent positioning of one of the NH of the amino group of **S18686** and one of the NH of the protonated piperazine of **5a** (see Figure 6F). No N1 MIFs superpositions are highlighted. DRY MIFs exhibited relatively large areas of overlap. 

Then, we found a lesser compatibility of the MIFs. Compound **9a** (Glob-Prod = 0.2692) exhibited no superposition of O MIFs, but a certain overlap of N1 fields, due to a similar positioning of **S18686** oxazoline nitrogen, and the pyridine one **9a** (Figure S1A). DRY MIFs overlap quite widely in the front area of the molecules. Compound **10a** (Glob-Prod = 0.2463) showed no significant MIF superpositions. In particular, the O MIF deriving from the protonated amine of the candidate partially overlaps with the N1 MIF generated by the oxazoline nitrogen of **S18686** (Figure S1B).

Similarly, the two last-scored compounds, **7a** (Glob-Prod = 0.1051) and **8a** (Glob-Prod = 0.2435) (Figure S1C,D), showed very small or no superpositions of HBD/A or DRY MIFs with the template, respectively, which is the reason for the low positioning of these candidates in the score list. In Figure S2 an overall perspective of the main MIFs shared by the best-scored candidate **1a** and the lowest analogue **7a**, with respect to the template **S18616**, was reported.

While ligand-based studies allowed us to compare the main steric and electrostatic features of **1a**–**10a** with those of **1**, taken as reference agonist, molecular docking studies of the in-house pyrimidinone-benzimidazole series were exploited to assess the ability of this chemo-type to simulate the docking mode of the **1**–**37** agonists.

Molecular docking studies of compounds **1a**–**10a** have been performed, relying on the AlphaFold protein structure. Details of the molecular docking calculations are shown in the experimental section. The obtained scoring functions for the pyrimidinone-benzimidazole-based compounds are listed in Table S6. 

As shown in Table 3, a perspective of the chemical structure of **1a**–**10a [59]** and of the predicted ΔG value of each best-scored protein–ligand complex is listed. The corresponding hTAAR1 EC_50_ values have been added, based on the following biological assays. 

The derived docking poses of only the most promising TAAR1 agonists based on the obtained scoring functions, with respect to the control compound **S186161**, are discussed. As a result, the putative most interesting derivatives were **1a**–**3a**, exhibiting S values < −3.0000 Kcal/mol, while **4a**–**6a** spanned from −3.1141 to −2.6083 Kcal/mol. Conversely, the analogues **7a**–**10a** were predictive as poorly active/inactive exhibiting S values > −1.4568 Kcal/mol. On this basis, the docking modes of only **1a**–**6a** are discussed as follows.

According to our calculations, the area of interaction of most of the aforementioned **1a**–**6a** is the same of the reference one **S18616 1**.

The choice of a piperazine group in R^1^ in tandem with a small hydrophobic chain in R is predicted as the most beneficial substitution involving the pyrimidinone-benzimidazole core, as shown by **1a** and **2a**, based on their obtained scoring functions.

**Figure 7:**
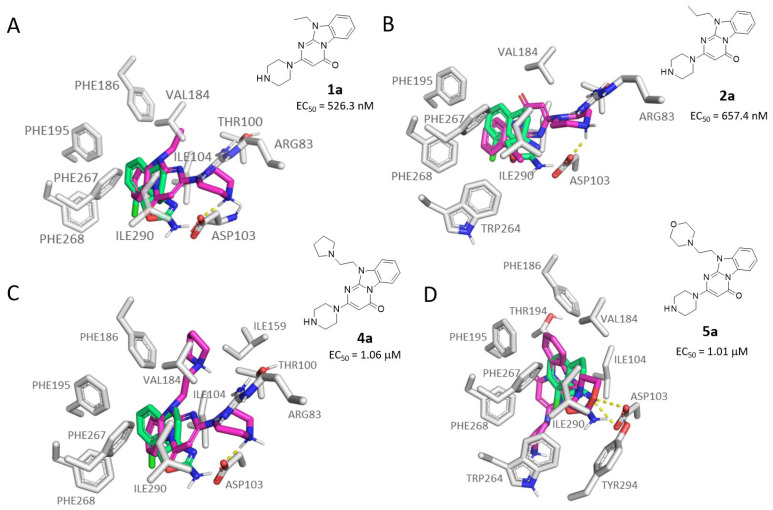
Molecular docking pose of compound **1a** (C atom; violet) (**A**), **2a** (C atom; violet) (**B**), **4a** (C atom; violet) (**C**), and **5a** (C atom; violet) (**D**) within the hTAAR1 binding site. The docking pose of the reference compound **S18616 1** [43] is reported (C atom; light green). The most important residues are shown and labeled. H-bonds are highlighted as dashed yellow lines. The chemical structures of compounds **1a**, **2a, 5a,** and **6a** [59] are reported.

In the compound **1a** the tri-cyclic core established non-polar interactions with residues PHE186, THR194 (methyl), ILE104, PHE268, PHE267, PHE195, VAL184, and ILE290, with the N-methyl substituent inserted within PHE186, THR100, and VAL184 sidechains (Figure 7A). The piperazine NH established an H-bond with ASP103 carboxylic group, while the aliphatic part of the ring contacts ILE290, VAL184, THR100 (methyl), and HIS99.

For compound **2a**, the orientation of the core is more similar to the **1a** case, with the phenyl ring on the left side, and the cyclo-amide on the right. However, the result is that the compound is upside-down, as the N-aliphatic substituent is pointing downwards, while the carbonyl is pointing upwards (Figure 7B). The core of the compound established non-polar interactions with ILE104, THR194, VAL184, PHE268, PHE267, and PHE195 (offset π-stacking). London forces were present between the iPr moiety of the ligand and residues PHE267, ILE290, and TRP264. The piperazine ring again made one H-bond with ASP103, while the non-polar part of the ring interacted with residues VAL184, THR100, HIS99, ILE290.

The introduction of an aromatic pendant at the N (10) atom led to the derivative **3a** and **6a**, exhibiting the benzyl and the pyridinil-methyl-groups in R, respectively.

In case of compound **3a**, the interaction pattern was coherent with the previous case, contacting PHE195, ILE104, PHE268, TRP264, with the benzyl inserted among residues PHE267 (staggered π-interaction), VAL184, THR271, PHE195 (T-shaped π-stacking), and PHE186. The protonated piperazine ring established one H-bond with ASP103, in addition to non-polar contacts with residues PHE267, ILE290, TYR294, VAL184 (see Figure S3A).

Regarding **6a**, the tri-cyclic core orientation slightly differed from the previous compounds (**1a** and **3a**). In this case, in fact, the phenyl group was positioned in the upper part of the binding pocket, within residues PHE186, THR194, PHE195 (staggered π-interaction), THR271, VAL184, and PHE267, while the rest of the core contacted residues PHE268, and PHE199 (see Figure S3B). Interestingly, the chlorine atom was inserted in a volume delimited by residues PHE199, SER108, and SER107, as in the case of some of the amino-oxazoline derivatives previously examined. When a pyridine ring was introduced in place of a piperazine, no H-bonds were possible with residue ASP103. However, this moiety established several other non-polar interactions with residues VAL184, ILE290, ILE104, TRP264, and PHE267 (staggered π-interaction). Interestingly, the pyridine ring was also involved in an intra-molecular T-shaped π-stacking with the phenyl ring of the tri-cyclic system.

Finally, the introduction of heteroalkyl-based chains in N (10) led to the pyrimidinone benzimidazoles **4a** and **5a**, bearing the pyrrolidine and morpholine substituents in R, respectively.

As regards compound **4a**, the piperazine moiety established one H-bond with ASP103 (Figure 7C). 

The tri-cyclic core established non-polar interactions with the surrounding residues (ILE290, PHE267, PHE268, PHE195, ILE104). The ethyl-N-cyclopentyl- moiety protrudes towards ASN164, and established non-polar contacts with residues PHE186, ILE159, VAL184, THR100.

The orientation of the **5a** core allowed the morpholine ring to be H-bonded with TYR294 (through the oxygen atom) and ASP103 (by means of the protonated tertiary amine) (see Figure 7D). In addition, this moiety also established non-polar interactions with VAL184, PHE267, TRP264, ILE290. The tri-cyclic core interacted with the surrounding residues (ILE104, THR194, PHE186, PHE267, and PHE195 (offset π-interaction with the phenyl ring of the ligand). The piperazine ring was inserted in a hydrophobic sub-pocket, interacting with PHE268, TRP264, ILE111, and PHE260.

### 2.3. Biological Evaluation of Compounds **1a**–**10a** as Novel TAAR1 Agonists

According to the results obtained via LBCM in tandem with those suggested by the previously cited structure-based results, we proceeded with the biological evaluation of the ten explored pyrimidinone-benzimidazoles **1a**–**10a**. While compounds **1a**–**6a** proved to be the most promising in silico, based on the previously cited computational studies, in vitro screening of the derivatives **7a**–**10a** has been also performed to elucidate the SAR of the series.

**Figure 8:**
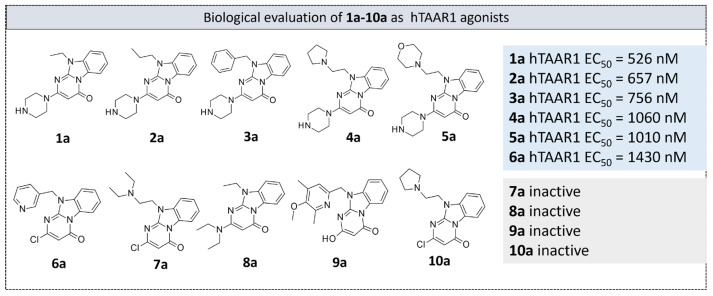
Chemical structure and hTAAR1 EC_50_ values of the herein screened agonists **1a**–**10a [59]**.

Thus, all the novel compounds **1a**–**10a** were tested at 10 μM to evaluate their potential hTAAR1 agonism ability (Figure 8), by an in vitro screening method based on the bioluminescence resonance energy transfer technique (BRET) (see the experimental section) [63]. 

The activity of these compounds was determined by means of HEK-293 cells transfected with hTAAR1, or empty vector as control, and a cAMP BRET biosensor. The applied positive control compound for agonism was tyramine hydrochloride (1 μM). The EC_50_ value of the positive control derivative was 41.6 nM under the experimental conditions.

For the compounds that were confirmed as active, a dose-response experiment was performed using concentrations ranging from 10 nM to 100 μM to derive the related EC_50_ values. All the compounds displayed an Emax > 85% at m/hTAAR1, and thus they were regarded as full agonists.

Among the active compounds **1a**–**6a**, the piperazine-containing derivatives **1a**–**5a** (hTAAR1 EC_50_ = 525–1060 nM) turned out to be more potent than **6a** (hTAAR1 EC_50_ = 1430 nM). In particular, the choice of the piperazine basic ring in the presence of a small hydrophobic chain in N (10) led to the most promising pyrimidinone-benzimidazoles **1a**–**2a** (hTAAR1 EC_50_ = 526–657 nM), endowed with comparable or higher potency values with respect to the 18% of the oxazoline derivatives **1**–**37** previously reported. These effective structural variations were in accordance with the reported molecular docking poses revealing beneficial Van der Waals contacts and π-π stacking with the hTAAR1 cavity. Removing the piperazine ring in R^1^ led to compounds **6a**–**10a**, most of them (**7a**–**10a**) being inactive as TAAR1 agonists.

The introduction of a flexible aromatic pendant in N (10) was well-tolerated, especially in the case of a maintained piperazine ring in R^1^. Accordingly, compound **3a** (hTAAR1 EC_50_ = 756 nM) displayed lower hTAAR1 EC_50_ values than **6a** (hTAAR1 EC_50_ = 1430 nM). Finally, including electron-rich atoms within the R substituents could impact the overall positioning of the compound, leading to mildly potent hTAAR1 agonists (see **4a**, **5a**; hTAAR1 EC_50_ = 1010–1060 nM). 

Based on the obtained biological assays, a perspective of the calculated ADMET properties for the most promising pyrimidinone-benzimidazole derivatives has been performed.

Nowadays, in silico prediction of absorption, distribution, metabolism, excretion, and toxicity (ADMET) properties is thought to be a valuable supporting tool in medicinal chemistry and these techniques are widely exploited in the literature [34,68,69].

Herein, ADMET properties explaining the drug-like profile of compounds **1a**-**6a** and **S186116** have been predicted, via the ACD/Lab Percepta platform [70] and SwissADME website [71]. Both of them are well-known tools applied in the prediction of pharmacokinetic properties [72,73,74,75,76,77,78].

Based on Veber’s [79] and Lipinski’s rules [80], the following descriptors have been calculated: (i) logarithmic ratio of the octanol–water partitioning coefficient (cLogP), (ii) molecular weight (MW) of compounds, (iii) H-bonding acceptor number (HBA), and H-bonding donor moieties (HBD), (iv) number of rotatable bonds (nRot_bond), (v) topological polar surface area (TPSA) (see Table 4). 

All the herein explored TAAR1 agonists **1a**–**6a** and the reference **S18616 1** fulfilled most of Lipinski’s rule and Veber’s rule. 

Further ADME parameters have been taken into account such as: (i) human intestinal absorption (HIA), estimation of the plasmatic protein binding event (% PPB), volume of distribution (Vd), ligand affinity toward human serum albumin (LogKa HSA) and of putative oral bioavailability, as a percentage (F %) (see Table 4).

Based on the above, among the newly developed TAAR1 agonists **1a**–**6a** the most interesting, **1a** (EC_50_ = 526 nM), featuring a small alkyl chain in R, showed quite comparable MW and TPSA values to those of the reference ligand **S18161**. On the other hand, it was endowed with modest HIA and F% values, if compared to the **S18161**.

The choice of H-bonding features in R (**4a**, **5a**; EC_50_ = 1010–1060 nM) led to reduced LogP values and increased the TPSA ones. This kind of variation impaired the HIA levels as well as the predicted F% parameter. Excessive TPSA values are also predicted to compromise blood–brain barrier permeability, making **5a** probably inactive in the CNS. 

Conversely, the introduction of the benzyl substituent in R, as shown by **3a** (EC_50_ = 756 nM), allowed an increase in the LogP value with respect to the alkyl-based analogues **1a**–**2a**, turning in ameliorated HIA and F% values. Indeed, **3a** was endowed with quite comparable F values with respect to **S18616**. Accordingly, the pyridine containing- derivative **6a** (EC_50_ = 1430 nM) also exhibits high HIA and modest F% values, being predicted as able to pass the blood–brain barrier.

In silico evaluation of toxicity properties in terms of cytochrome inhibition and of lethal dose via mouse oral administration have been explored as well as PAINS (Pan-Assay Interference Structures) analysis (see Table S7). The results pointed out no cytochrome inhibition events or PAINS ability. Finally, additional in silico evaluations of the most promising compounds **1a**–**3a** have been performed via SwissTarget [71,81,82,83,84], suggesting a prominent role as GPCRs or enzyme-targeting compounds for the three aforementioned pyrimidinone-benzimidazoles **1a**–**3a** (see Figure S4). This information is in agreement with the results herein reported regarding their TAAR1 agonism ability and also for the previously described mechanism of action as human platelet aggregation inhibitors via phosphodiesterase inhibition [59].

## 3. Discussion

The development of TAAR1 ligands represents an intriguing strategy for the treatment of several disorders including schizophrenia, depression, attention deficit hyperactivity disorder, Parkinson’s disease, addiction, and metabolic diseases [22]. As shown in the literature, repositioning approaches involving different series of GPCR-targeting compounds have been explored [23,24,25], such as dopaminergic, serotonergic, and adrenergic drugs, such as the agonist **S18616**.

Recently, structural variations of the main **S18616** tricyclic ring were afforded, leading to different series of more selective TAAR1 ligands featuring the amino-oxazoline main core [36,37,38]. 

Compounds **1**–**37,** previously disclosed in the literature and herein investigated in silico, displayed effective substitutions linked to the main five-membered ring, such as oxazoline-based phenyl-, benzyl-, phenyl (amino)alkyl-containing derivatives. Among them, a number of phenyl (hetero)alkyl-containing compounds (**5**–**21**; EC_50_ = 9–2260 nM) and phenyl-based (**22**–**37**; EC_50_ = 11–10,000 nM) compounds showed promising hTAAR1 EC_50_ values.

Herein, we explored via molecular docking studies the effectiveness of these structural variations in terms of TAAR1 targeting ability, thanks to the protein AlphaFold model. The results pointed out the relevant role played by aromatic cores linked to the oxazoline ring, being also endowed with H-bonding features. The introduction of branched and/or folded groups was promising, being in accordance with the pivotal role played by the TAAR1 agonist “Y-shape” conformation [62,64,65].

In particular, halogen and aliphatic substitutions on the additional phenyl ring exhibited complex effects, and the combination of substituents seems relevant. For the compounds without a linker, *p*-substitution alone was not very well tolerated, but activity was restored when it was combined with *o*- or *m*-substitutions. In the case of fluorine, *meta*-substitution alone was not tolerated, but further *o*-methyl-substitution led to excellent activity (compound **37**). For compounds bearing a linker, the information on phenyl substitution was not very abundant, being that unsubstituted rings exhibited good activity. 

In terms of protein–ligand interactions, the key feature for successful compounds was the unperturbed establishment of the key H-bond between the amine-oxazoline moiety and ASP103, together with at least one hydrophobic moiety. In particular, aromatic rings can establish π-interactions, in addition to London forces, due to the presence of several aromatic residues in the TAAR1 pocket. The presence of an additional steric hindrance in the sub-pocket formed by SER107, SER108, and PHE268 was favorable. 

On this basis, following molecular modeling, studies have been developed based on LBCM and SBCM to explore compounds **1a**–**10a** [59] as putative TAAR1 agonists (the chemical structures in Table S2). As regards the structure-based results, the flat and mainly aromatic tricyclic ring of all the pyrimidinone-benzimidazole derivatives **1a**–**10a** mimics the main core of the reference compound **S18616**, compounds **1a**–**3a** being predicted as the most promising as putative hTAAR1 ligands. In particular, the choice of a piperazine group in R^1^ in tandem with a small hydrophobic chain in R allowed the compounds to exhibit key contacts with the protein cavity, such as: (i) non-polar interactions with residues PHE186, THR194, ILE104, PHE268, PHE267, PHE195, VAL184, and ILE290, and (ii) an H-bond with the ASP103 carboxylic group.

The results obtained by the concomitant ligand-based studies were in agreement with the following biological assays. Indeed, the best-scored compounds **1a**–**3a** (hTAAR1 EC_50_ = 526.3–756.80 nM) were properly predicted as the most effective TAAR1 agonists within the pyrimidinone-benzimidazole series, exhibiting the highest Glob-Prod score values (Glob-Prod = 0.3154–0.3499). The calculated **1a**–**3a** MIFs were in good agreement with those featured by the template **S18616**, thanks to the corresponding basic moieties and aromatic features. While the analogues **4a**–**6a** partially mimic the template MIFs due to the amine groups, the lowest-scored congeners **7a**–**10a** poorly simulated the reference MIFs. Accordingly, the following biological evaluation revealed **4a**–**6a** (hTAAR1 = 1010–1430 nM) as modest TAAR1 agonists. A perspective of this information pointed out the pyrimidinone-benzimidazole series as a novel chemo-type for the development of novel TAAR1-targeting compounds, exhibiting a new core if compared to the limited number of the so far explored TAAR1 agonists. 

Finally, in silico prediction of ADMET properties and PAINS (Pan Assay Interference structures) analysis gave further support to the most promising compounds **1a**–**3a** as drug-like derivatives. Among them, compound **3a** was endowed with the highest predicted LD_50_ values and oral bioavailability parameters. 

## 4. Materials and Methods

### 4.1. Computational Studies

All the studied compounds, including the oxazoline **1**–**37** and the pyrimidinone benzimidazoles **1a**–**10a,** were manually built by the MOE Builder program included in the MOE software [67] and assigned the prevalent protonation state at pH = 7.4 through the wash function. Ligand parametrization was carried out with the AM1 partial charges as calculation method. Energy minimization step has been performed by the Energy Minimize Program using MMFF94x forcefield of MOE and RMS (root mean square) gradient equal to 0.0001, being the root mean square gradient of the norm of the gradient times the square root of the number of (unfixed) atoms. This approach allowed us to build in silico a single low-energy conformation for each compound [67]. The tridimensional model of the hTAAR1 receptor has been retrieved via the AlphaFold protein model database [56], using the AF-Q96RJ0-F1 code. The model was processed by means of the “Structure Preparation” tool from MOE 2019.01 suite and refined via the “Protonate3D” tool as previously mentioned [61]. This allowed us to assign the most probable protonation state to each residue being the partial charges determined according to the AMBER10:EHT force field, included in the MOE software.

All the molecular docking simulations at the hTAAR1 AlphaFold protein model were performed by means of the DOCK tool included in the MOE software, via a template-based approach using the previously described **S18616**-TAAR1 complex [61]. 

This was developed applying the Alpha Triangle method and Affinity ΔG prediction as final scoring function to obtain from 50 to 10 best score conformers. Details of the template-based docking calculations as well as of the scoring functions are shown in our previous papers [85,86].

The LBCM studies were performed with FLAP2.2.1 Ligand-Based module [60,61], using as a template the minimized form of compound **S18686** [43], a well-known TAAR1 agonist endowed with high potency and a rigid scaffold. For each of the ten candidates, 25 conformers were generated starting from their optimized geometry (ligand preparation step previously described). Four probes were selected for the analysis, namely the H probe (expressing shape similarity), DRY (hydrophobic probe), O (HBA probe, corresponding to a carbonyl-group), and N1 (HBD probe, corresponding to amide NH). Briefly, the ligand is inserted in a grid with nodes at a defined distance from each other (here 0.75Å). Each probe is positioned in correspondence with the grid nodes, and the interaction energies with the ligand are calculated. The output of this analysis can be visualized as 3D-objects called Molecular Interaction Fields (MIFs), identifying areas of favored interactions between the ligand and the considered probe. For each molecule, several sets of quadruplets representing the most favorable interactions are extracted by the MIFs, enabling an easily-comparable representation. All the quadruplets of a molecule are stored together, representing a pharmacophore fingerprint of the molecule itself. Such quadruplets are used to generate different superimpositions to the template, and the Probe scores and Distance scores are produced. The Probe scores represent the degree of overlap of the MIFs of the test molecule and the template for each probe individually. The Distance score indicates the overall difference of probe score between the ligand and template. The product of the individual Probe scores gives the Glob-Prod global score, ranging from 0 (bad) to 1 (good) [60,87]. In the present case, the Glob-Prod score was considered for compound evaluation, as different types of interactions are expected based on the shape and electrostatic/steric properties of the reference compound **1**. Moreover, MIF information was used to visually compare the in-house set of ligands with the known active compound, the template (**S18686**) [43] In fact, the superposition of the same type of MIF between the target molecule and the template is regarded as sign of ligand similarity, enhancing the probability of giving a similar biological output [60].

### 4.2. Chemicals

Compounds **1a**–**10a** were synthesized as previously described [46]. The synthetic route to compounds **1a**–**5a** and **7a**, **10a** included the reaction of 2-aminobenzimidazole with proper alkyl halides in refluxing acetone in the presence of anhydrous K_2_CO_3_/ KOH to obtain the corresponding 1-substituted 2-aminobenzimidazoles. On the contrary, in the case of the pyridine-containing compounds **6a** and **9a**, 2-aminobenzimidazole was treated with an equimolar amount of EtONa in anhydrous EtOH to give the sodium salt derivative, prior to treatment with the proper alkyl halide. 

The 1-substituted 2-aminobenzimidazoles were then condensed with diethyl malonate in the presence of EtONa to afford good yields of crystalline sodium salts of the corresponding 10-substituted 2-hydroxypyrimido [1,2-*a*]benzimidazol-4(10*H*)-ones. These compounds were subsequently treated with excess refluxing POCl_3_ affording the corresponding 2-chloroderivatives (such as **6a**, **7a**). The following reaction of 2-chloroderivatives with excess piperazine in refluxing ethanol led to the desired 10-substituted 2-(1-piperazinyl) pyrimido [1,2-*a*]benzimidazol-4(10*H*)-ones (such as **1a**–**5a**).

### 4.3. Biological Evaluation of Compounds **1a**–**10a** as TAAR1 Agonists

#### Bioluminescence Resonance Energy Transfer (BRET) Measurement

BRET screening has been described in detail elsewhere [63,88,89,90,91,92,93,94]. Briefly, human embryonic kidney 293 cells (HEK293T; ATCC CRL-11268) were transiently co-transfected with plasmids encoding hTAAR1 and a cAMP BRET biosensor using Lipofectamine^®^ 2000 reagent (ThermoFisher, Waltham, MA, USA), and then plated in 96-well white opaque microplates (Corning, Corning, NY, USA) at a density of 50 × 104 cells per well. After 24-h incubation, culture medium was removed and 70 µL of Hank’s balanced salt solution containing calcium and magnesium was added to each well, followed by the addition of 10 µL 200 mM 3-isobutyl-1-methylxanthine solution (Sigma, St. Louis, MO, USA), a phosphodiesterase inhibitor, and 10 µL 50 µM coelenterazine-h solution (Promega, Madison, WI, USA), as luciferase substrate. All tested compounds were dissolved in DMSO to yield 10 mM stock solutions. After 10 min incubation, either 10 µL of vehicle or 10× of the concentrated solution of compound to be tested (10 µM final concentration) was added. After an additional 5-min incubation, luminescence readings were collected using a Mithras LB943 multimodal plate reader (Berthold Technologies, Wildbad, Germany). The BRET signal was determined by calculating the ratio of the light emitted at 505 to 555 nm to the light emitted at 465 to 505 nm. To confirm the specificity of positive responses, parallel screening on cells transfected with empty core vector was carried out. For active compounds, separate dose–response experiments were performed in order to calculate the EC_50_ values. Curves were fitted by applying non-linear regression models in GraphPad Prism 6 (GraphPad Software, San Diego, CA, USA). Data are representative of four independent experiments and are expressed as means (errors in EC_50_ are within 10%).

The prediction of all the reported ADMET parameters was developed by means of the Advanced Chemistry Development (ACD) Percepta platform [70]. The software prediction is performed based on the software implemented training libraries, which include experimentally determined pharmacokinetic and safety properties for different series of compounds. Prediction of PAINS (Pan Assay Interference structures) was derived via the SwissADME website [71].

## 5. Conclusions

Recently, a number of combined structure-based studies and ligand-based studies have been attempted to explore the selectivity and species-specificity profile of different series of TAAR1 ligands. 

Herein, based on the hTAAR1 AlphaFold protein model, we explored the effectiveness of structural variations involving the main scaffold of the conformationally locked hTAAR1 agonist **S18616.** The analogues **1**–**37** have been investigated via molecular docking studies, prior to proceeding with the biological evaluation of a series of *in-house* pyrimidinone-benzimidazole derivatives (**1a**–**10a**). Based on the combined LB- and SBCM results, **1a**–**10a** have been assayed as putative TAAR1 agonists. Final biological evaluation revealed the piperazine-containing derivatives **1a**–**3a** (hTAAR1 EC_50_ = 526.3–657.4 nM) as the most promising of the series and allowed us to confirm the reliability of the computational studies. Then, in silico prediction of the compounds ADMET properties suggested the future development of the pyrimidinone-benzimidazole scaffold in the search for novel effective hTAAR1 agonists.

## Figures and Tables

**Table 1:** Chemical structure and biological activity as hTAAR1 agonists (EC_50_ values) of compound **1** and of compounds (Comp.) **2**–**21**, featuring a flexible linker between the oxazoline ring and the terminal phenyl group [38,43]. The predicted ΔG value of each best-scored protein–ligand complex has been reported, as calculated in terms of molecular docking final scoring function (S, as Kcal/mol).

Comp.	Chemical Structure	EC_50_ (nM)	S	Comp.	Chemical Structure	EC_50_ (nM)	S
**1**	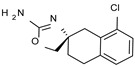	15	−6.8545	**12**	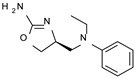	59	−6.1163
**2**	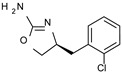	154	−6.2525	**13**	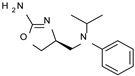	140	−5.1443
**3**	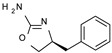	330	−6.1549	**14**	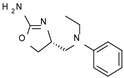	230	−6.3802
**4**	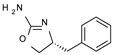	2900	−6.7972	**15**	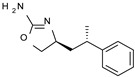	1540	−4.6935
**5**	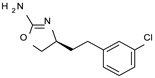	18	−6.8864	**16**	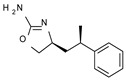	730	−7.2651
**6**	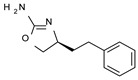	27	−7.1321	**17**	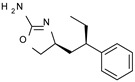	2260	−5.9924
**7**	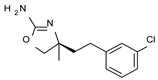	330	−5.6156	**18**	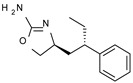	18	−5.8995
**8**	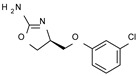	270	−6.1963	**19**	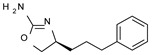	27	−5.8673
**9**	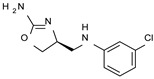	580	−4.9034	**20**	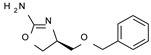	360	−5.1471
**10**	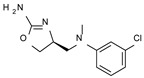	27	−6.5934	**21**	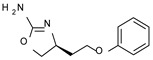	9	−7.1321
**11**	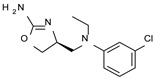	29	−6.5403				

**Table 2:** Chemical structure and biological activity as hTAAR1 agonists (EC_50_ values) of compounds (Comp.) **22**–**37**, as phenyl-based derivatives [38,43]. The predicted ΔG value of each best-scored protein–ligand complex has been reported, as calculated in terms of molecular docking final scoring function (S, as Kcal/mol).

Comp.	Chemical Structure	EC_50_ (nM)	S	Comp.	Chemical Structure	EC_50_ (nM)	S
**22**	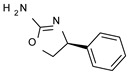	67	−6.2967	**30**	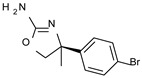	41	−5.7542
**23**	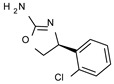	23	−6.6376	**31**	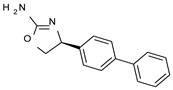	2670	−5.2125
**24**	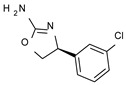	21	−6.7660	**32**	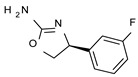	490	−5.6820
**25**	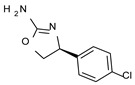	143	−6.0587	**33**	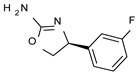	67	−6.7540
**26**	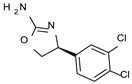	31	−6.5172	**34**	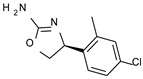	11	−6.9372
**27**	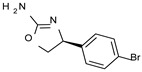	150	−5.9241	**35**	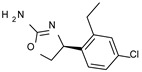	26	−6.4499
**28**	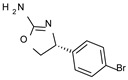	>10,000	−5.0876	**36**	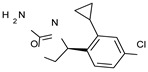	12	−6.0151
**29**	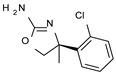	165	−5.5681	**37**	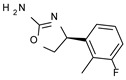	17	−6.6828

**Table 3:** Chemical structure and biological activity as hTAAR1 agonists (EC_50_ values) of the in-house compounds (Comp.) **1a**–**10a [59]**. The predicted ΔG value of each best-scored protein–ligand complex has been reported, as calculated in terms of molecular docking final scoring function (S, as Kcal/mol).

Comp.	Chemical Structure	EC_50_ (nM)	S	Comp.	Chemical Structure	EC_50_ (nM)	S
**1a**	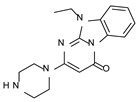	526	−3.1695	**6a**	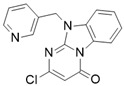	1430	−2.9174
**2a**	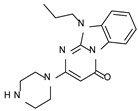	657	−3.1254	**7a**	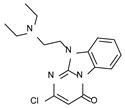	inactive	3.9286
**3a**	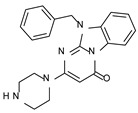	756	−3.1408	**8a**	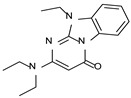	inactive	0.4922
**4a**	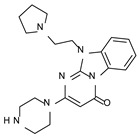	1060	−2.6083	**9a**	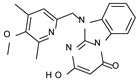	inactive	−1.4568
**5a**	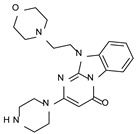	1010	−3.1141	**10a**	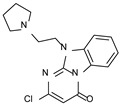	inactive	0.4922

**Table 4:** Calculated parameters related to Lipinski’s rules, Veber’s rules, and absorption/distribution properties, referred to the compounds (Comp.) **1a**–**6a** [59] and to the reference **S18616** [43], are reported. The corresponding chemical structure and hTAAR1 EC_50_ values (EC_50_) are also shown. Compounds predicted as unable to pass the blood–brain barrier are listed in italics. Reliability index values for a number of descriptors are shown as R.I. Values higher than 0.30 are ranked as reliable by the Advanced Chemistry Development (ACD) Percepta platform (https://www.acdlabs.com/products/percepta-platform/, accessed on 8 April 2024) [70].

Comp.	MW ^a^	N. H-Bond Acceptor ^b^	N. H-Bond Donor ^c^	N. Rotatable Bonds ^d^	CLogP ^e^ (R.I. ≥ 0.45)	TPSA ^f^	LogBB ^g^	LogPS ^h^	HIA (%) ^i^	Vd (L/kg) ^j^	%PPB ^k^ (R.I. ≥ 0.40)	LogKa _HAS_^l^ (R.I. ≥ 0.30)	F% ^m^ (Oral)
**1a**	297.36	6	1	2	0.87	51.18	−0.58	−3.1	46	2.1	84.09	4.48	27.1
**2a**	311.38	6	1	3	1.21	51.18	−0.51	−2.9	52	2.3	84.54	4.49	30.7
**3a**	359.42	6	1	3	2.21	51.18	−0.53	−2.5	95	2.8	95.79	4.95	80.8
**4a**	366.46	7	1	4	0.82	54.42	−0.46	3.3	17	4.6	77.89	4.32	8.9
***5a***	382.46	8	1	4	0.52	63.65	−0.48	−3.5	29	3.2	75.84	4.23	15.7
**6a**	310.74	5	0	2	2.53	48.80	−0.77	−1.3	100	1.7	98.25	5.29	37.7
**S18616**	236.70	3	2	0	3.11	47.61	0.56	−1.4	100	3.2	71.82	3.49	99.3
**Chemical structure**													
**1a** 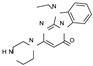 EC_50_ = 526 nM		**2a** 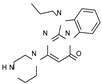 EC_50_ = 657 nM		**3a** 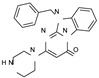 EC_50_ = 756 nM		**4a** 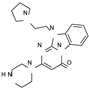 EC_50_ = 1060 nM		**5a** 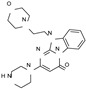 EC_50_ = 1010 nM		**6a** 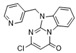 EC_50_ = 1430 nM		**S18616** 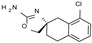 EC_50_ = 15 nM	

^a^ Molecular weight; ^b^ Number of H-bond acceptors; ^c^ Number of H-bond donors; ^d^ Number of rotatable bonds; ^e^ Logarithmic ratio of the octanol–water partitioning coefficient; ^f^ Topological polar surface area; ^g^ Extent of brain penetration based on ratio of total drug concentrations in tissue and plasma at steady-state conditions; ^h^ Rate of brain penetration. PS represents Permeability-Surface area product and is derived from the kinetic equation of capillary transport; ^i^ HIA represents the human intestinal absorption, expressed as percentage of the molecule able to pass through the intestinal membrane; ^j^ prediction of Volume of Distribution (Vd) of the compound in the body; ^k^ estimation of the plasmatic protein binding event (%PPB); ^l^ ligand affinity toward human serum albumin; ^m^ Percentage oral bioavailability.

## Data Availability

The data are contained within the article.

## Supplementary Materials

The following supporting information can be downloaded at https://www.mdpi.com/article/10.3390/molecules29081739/s1: Figure S1: MIF Comparison of the template **S18686** and of the candidates **9a**, **10a**, **8a,** and **7a**; Figure S2: Common MIFs between the template **S18686** and the candidates **1a** and **7a**; Figure S3: Molecular docking pose of compound **3a** (C atom; violet) (A) and **6a** (C atom; violet) (B) within the hTAAR1 binding site; Figure S4: Prediction of putative off-target preferences featured by **1a**–**3a**; Table S1: Chemical structure and biological activity as hTAAR1 agonists of compounds **1**–**37**; Table S2: Chemical structure of in-house compounds **1a**–**10a**; Table S3: Ten top-scored docking positioning of **1**–**37** at the hTAAR1; Table S4: The calculated scoring function Glob-Prod values based on the MIFs H, N1, DRY, O are reported (compounds **1a**–**10a** as candidates, **S18616** being the template); Table S5: The calculated scoring function Glob-Prod values based on the MIFs H, N1, DRY, O are reported (compounds **2**–**37** as candidates, **S18616** being the template); Table S6: Ten top scored docking positioning of **1a**–**10a** at the hTAAR1; Table S7: Calculated descriptors related to toxicity properties, cytochrome inhibition, and lethal dose via mouse oral administration have been explored for **1a**–**6a** and for the reference **S18616**.
