# Supplementary material for: Discovery of a Novel Chemo-Type for TAAR1 Agonism via Molecular Modeling
Source: Molecules. 2024 Apr 11;29(8):1739. doi: 10.3390/molecules29081739 (PMC11052455; doi:10.3390/molecules29081739)
Supplement: Supplementary file 1 [file molecules-29-01739-s001.zip › molecules-2897672-supplementary.pdf]

SUPPORTING INFORMATION

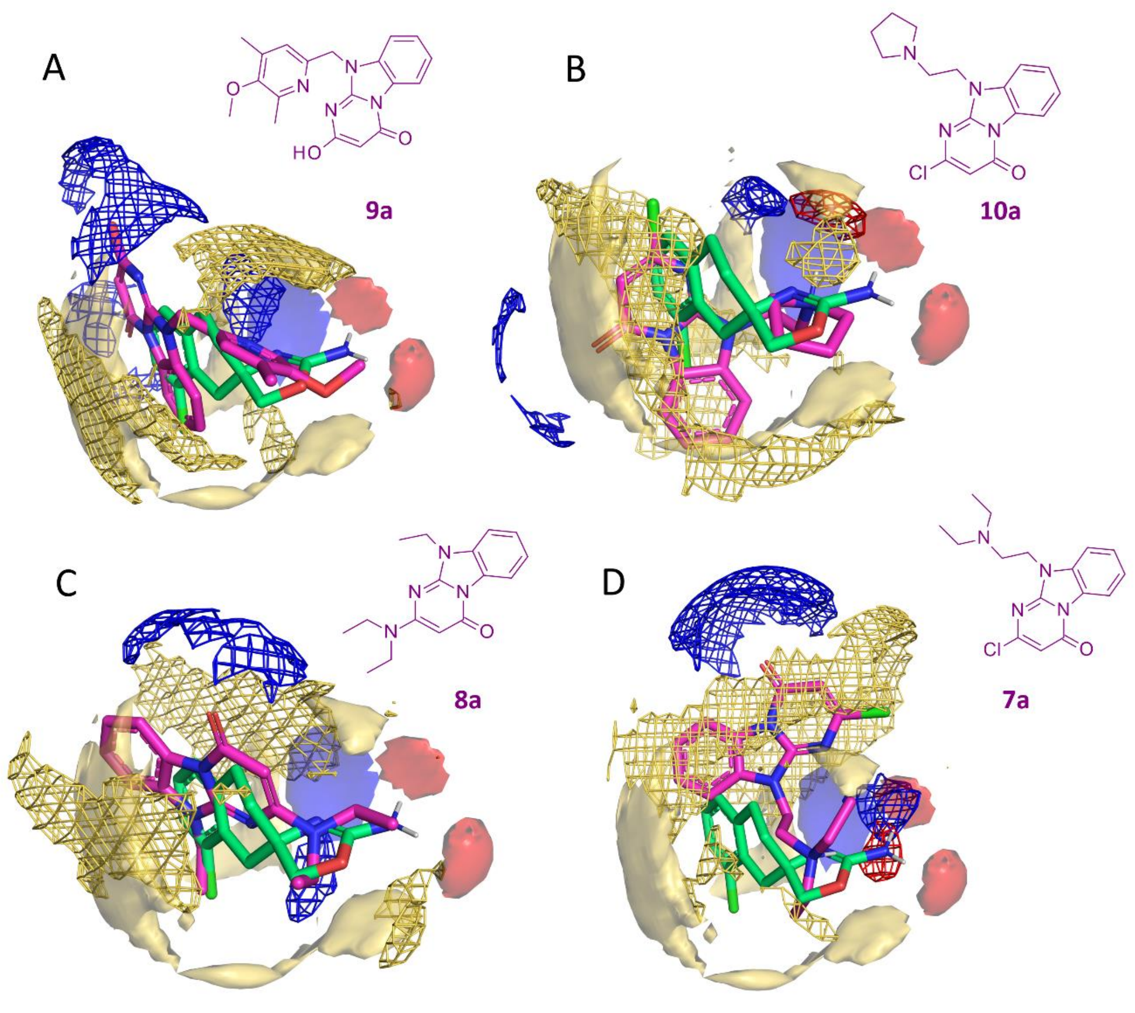

Figure S1. MIF Comparison of the template **S18686 1** [43] (C atom; green) MIFs (solid) and those (wireframes) of the candidates [59] (C atom; magenta). Blue: N1 MIF, red: O MIF, yellow: DRY MIF. The analysis is reported for compounds **9a** (A) **10a** (B), **8a** (C) and **7a** (D), based on the calculated Glob-Prod ranking (**9a**, Glob-Prod = 0.2692; **10a**, Glob-Prod = 0.2463; **8a**, Glob-Prod = 0.2435; **7a**, Glob-Prod = 0.1051).

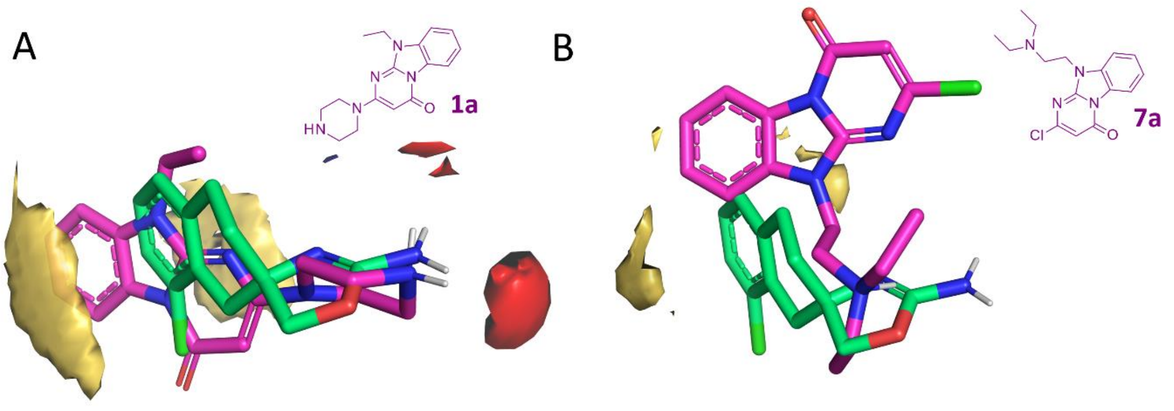

Figure S2. Common MIFs between the candidate and **S18686 1** [43]. Blue: N1 MIF, red: O MIF, yellow: DRY MIF. The analysis is reported for the top-scored compound **1a** (Glob-Prod = 0.3499) (A) and the lowest one **7a** (Glob-Prod = 0.1051) (B). For **1a** an almost large common MIF pattern is found (A). On the contrary, the common areas when considering **7a** are limited (B).

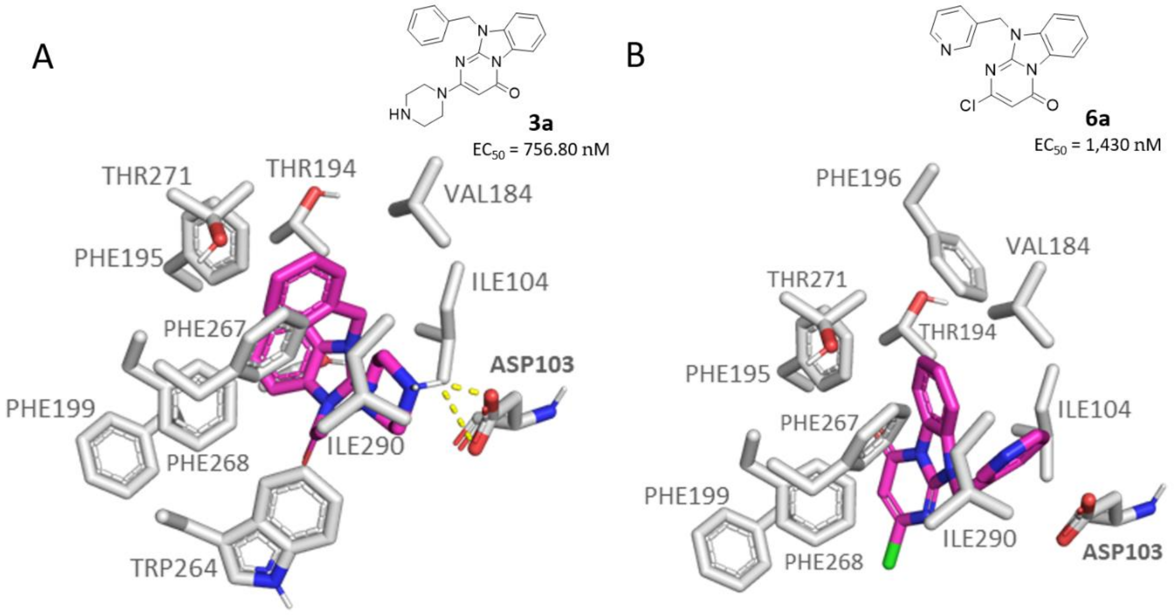

Figure S3. Molecular docking pose of compound **3a** (C atom; violet) (A) and **6a** (C atom; violet) (B) within the *h*TAAR1 binding site. The most important residues are shown and labeled. The chemical structure of the two compounds **3a** [59] and **6a** [59] is also reported.

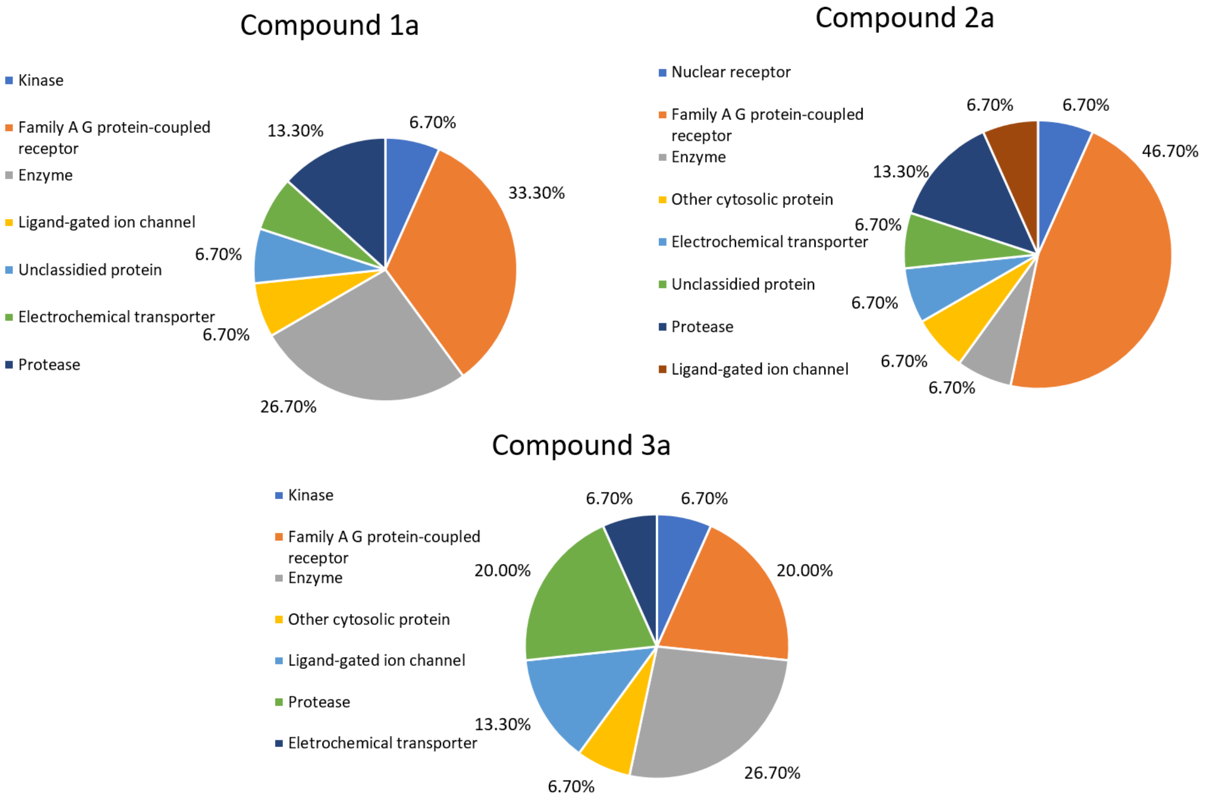

Figure S4. Prediction of putative off-targets preferences featured by **1a**-**3a** [59]. The reported in silico evaluation was performed thanks to SwissTarget website [60].

Table S1. Chemical structure and biological activity as *h*TAAR1 agonists of compounds **1-37** [38, 43].

**Compound**	**SMILE structure**	**Chemical structure**	***h*TAAR1 EC$_{50}$ (nM)**
**S18616 (1)**	Clc1c2CC3(N=C(OC3)N)CCc2ccc1	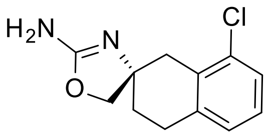	15
**2**	Clc1ccccc1C[C@@H]1N=C(OC1)N	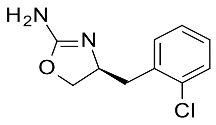	154
**3**	O1C[C@@H](N=C1N)Cc1ccccc1	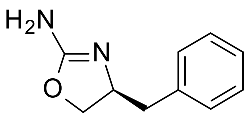	330
**4**	O1C[C@H](N=C1N)Cc1ccccc1	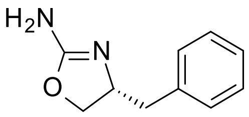	2900
**5**	Clc1cc(ccc1)CC[C@@H]1N=C(OC1)N	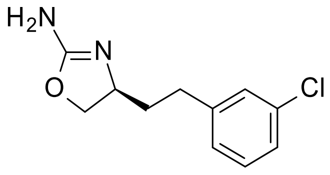	18

**6**	O1C[C@@H](N=C1N)CCc1ccccc1	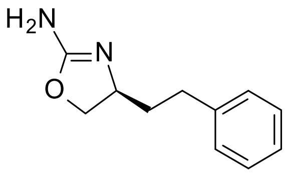	27
**7**	Clc1cc(ccc1)CC[C@@]1(N=C(OC1)N)C	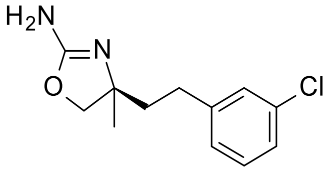	330
**8**	Clc1cc(OC[C@@H]2N=C(OC2)N)ccc1	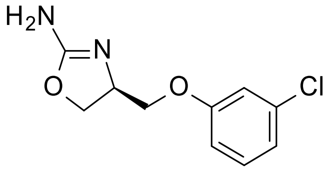	270
**9**	Clc1cc(NC[C@@H]2N=C(OC2)N)ccc1	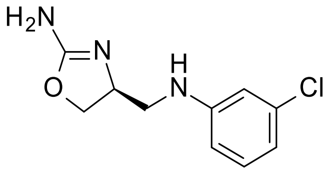	580
**10**	Clc1cc(N(C[C@@H]2N=C(OC2)N)C)ccc1	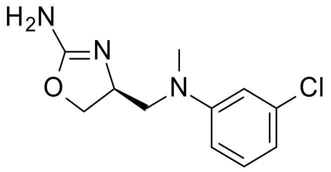	27
**11**	Clc1cc(N(C[C@@H]2N=C(OC2)N)CC)ccc1	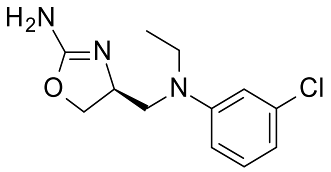	29
**12**	O1C[C@@H](N=C1N)CN(CC)c1ccccc1	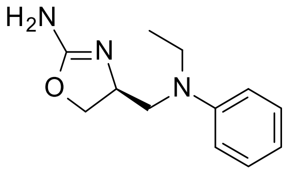	59
**13**	O1C[C@@H](N=C1N)CN(C(C)C)c1ccccc1	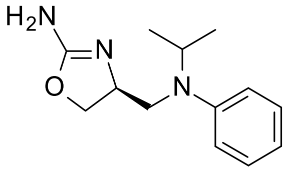	140
**14**	O1C[C@H](N=C1N)CN(CC)c1ccccc1	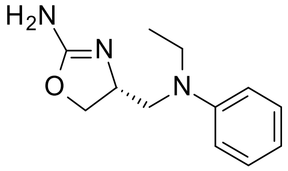	230
**15**	O1C[C@@H](N=C1N)C[C@H](C)c1ccccc1	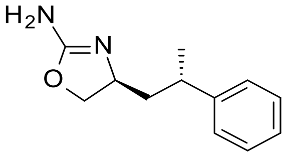	1540

**16**	O1C[C@@H](N=C1N)C[C@@H](C)c1ccccc1	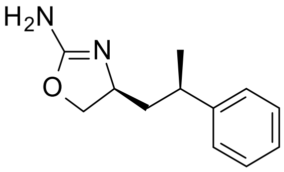	730
**17**	O1C[C@@H](N=C1N)C[C@@H](CC)c1ccccc1	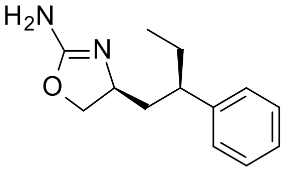	2260
**18**	O1C[C@@H](N=C1N)C[C@H](CC)c1ccccc1	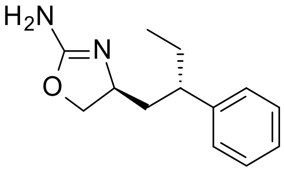	18
**19**	O1C[C@@H](N=C1N)CCCc1ccccc1	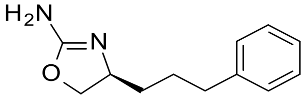	27
**20**	O1C[C@@H](N=C1N)COCc1ccccc1	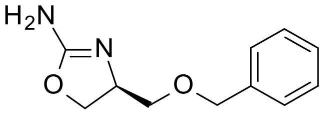	360
**21**	O1C[C@@H](N=C1N)CCOc1ccccc1	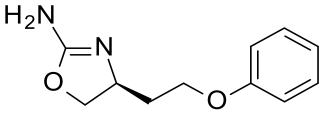	9
**22**	O1CC(N=C1N)c1ccccc1	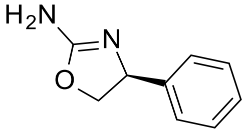	67
**23**	Clc1ccccc1C1N=C(OC1)N	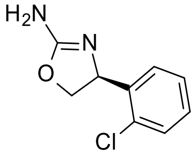	23
**24**	Clc1cc(ccc1)C1N=C(OC1)N	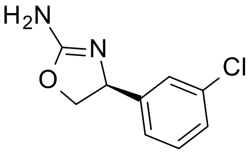	21
**25**	Clc1ccc(cc1)C1N=C(OC1)N	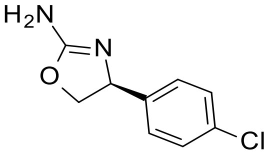	143
**26**	Clc1cc(ccc1Cl)C1N=C(OC1)N	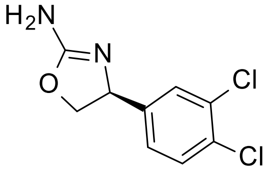	31

**27**	Brc1ccc(cc1)C1N=C(OC1)N	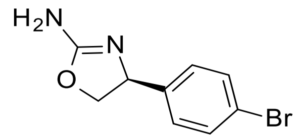	150
**28**	Brc1ccc(cc1)[C@H]1N=C(OC1)N	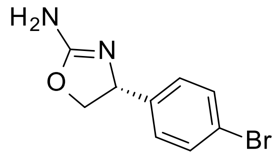	>10000
**29**	Clc1ccccc1[C@@]1(N=C(OC1)N)C	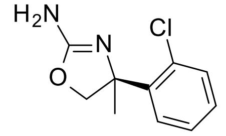	165
**30**	Brc1ccc(cc1)[C@@]1(N=C(OC1)N)C	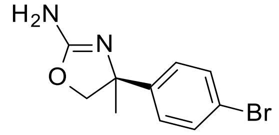	41
**31**	O1C[C@@H](N=C1N)c1ccc(cc1)-c1ccccc1	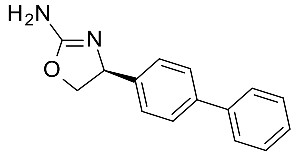	2670
**32**	Fc1cc(ccc1)[C@@H]1N=C(OC1)N	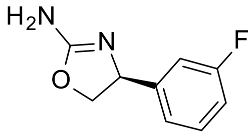	490
**33**	O1C[C@@H](N=C1N)c1ccccc1C	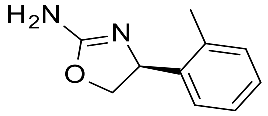	67
**34**	Clc1cc(C)c(cc1)[C@@H]1N=C(OC1)N	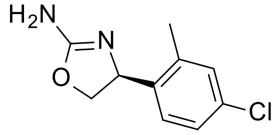	11
**35**	Clc1cc(CC)c(cc1)[C@@H]1N=C(OC1)N	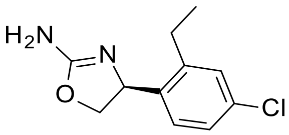	26
**36**	Clc1cc(C2CC2)c(cc1)[C@@H]1N=C(OC1)N	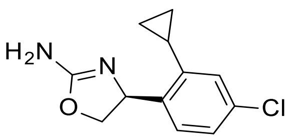	12
**37**	Fc1cccc([C@@H]2N=C(OC2)N)c1C	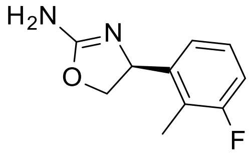	17

Table S2. Chemical structure of the in-house compounds **1a-10a** [59].

Compound	SMILE structure	Chemical structure

**1a**	O=C1N2c3c(N(C2=NC(N2CCNCC2)=C1)CC)cccc3	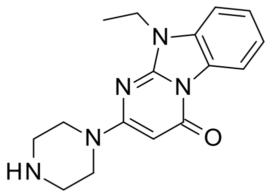
**2a**	O=C1N2c3c(N(C2=NC(N2CCNCC2)=C1)CCC)cccc3	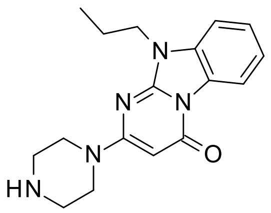
**3a**	O=C1N2c3c(N(C2=NC(N2CCNCC2)=C1)Cc1ccccc1)cccc3	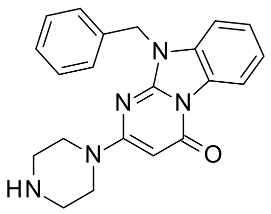
**4a**	O=C1N2c3c(N(C2=NC(N2CCNCC2)=C1)CCN1CCCC1)cccc3	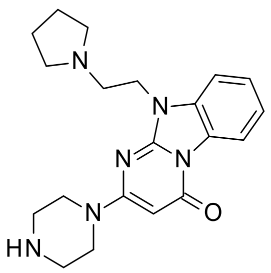
**5a**	O1CCN(CC1)CCN1c2c(N3C1=NC(N1CCNCC1)=CC3=O)cccc2	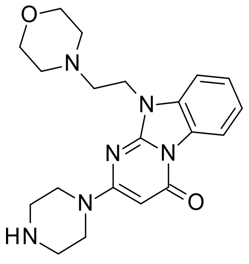
**6a**	ClC=1N=C2N(c3c(N2Cc2cccnc2)cccc3)C(=O)C=1	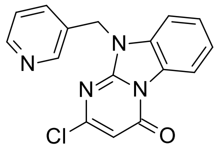
**7a**	ClC=1N=C2N(c3c(N2CCN(CC)CC)cccc3)C(=O)C=1	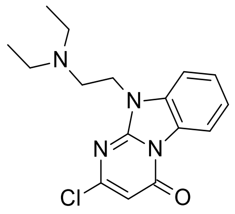
**8a**	O=C1N2c3c(N(C2=NC(N(CC)CC)=C1)CC)cccc3	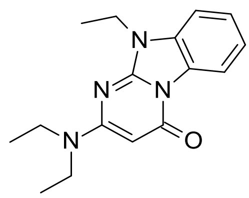

**9a**	O(C)c1c(cc(nc1C)CN1c2c(N3C1=NC(O)=CC3=O)cccc2)C	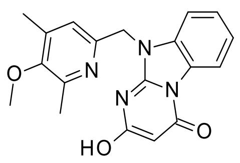
**10a**	ClC=1N=C2N(c3c(N2CCN2CCCC2)cccc3)C(=O)C=1	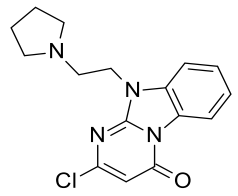

Table S3. Ten top scored docking positioning of **1-37** [38, 43] at the *h*TAAR1 (MOE software). The predicted ΔG value of each protein-ligand complex has been reported, as calculated in terms of final scoring function (S, as Kcal/mol). The top-scored pose for each compound is highlighted in cyan. The compounds EC_{50} values are also reported.

Compound	Pose	S	E_conf	E_place	E_score 1	E_refine	E_score 2
**1** *h*TAAR1 EC_{50} = 15 nM	1	-6.8545	-37.2407	-20.5108	-8.2982	36.9326	-6.8545
	2	-5.0428	-26.1434	-21.4527	-7.6237	56.3373	-5.0428
	3	-4.9404	-40.3592	-18.4309	-7.4393	37.3493	-4.9404
	4	-4.8130	-41.0371	-23.3400	-8.2540	26.4267	-4.8130
	5	-4.6694	-39.8789	-24.8717	-10.3595	40.1172	-4.6694
	6	-4.1973	-33.0527	-20.4493	-7.6808	55.0638	-4.1973
	7	-4.0104	-34.0839	-19.8069	-7.7222	71.2140	-4.0104
	8	-3.6697	-33.0988	-24.4046	-9.1082	49.9467	-3.6697
	9	-3.5519	-32.8718	-16.1752	-7.6066	56.6206	-3.5519
	10	-3.3840	-36.5861	-14.1440	-7.5789	51.6696	-3.3840
**2** *h*TAAR1 EC_{50} = 154 nM	1	-6.2525	-34.2820	-23.1189	-8.0869	63.0192	-6.2525
	2	-5.6656	-29.7123	-23.2903	-7.9338	52.7397	-5.6656
	3	-5.6250	-16.9957	-20.3914	-6.9026	59.4729	-5.6250
	4	-5.2571	-39.3765	-22.7332	-7.8917	50.8643	-5.2571
	5	-5.1724	-11.6383	-17.9664	-7.2081	60.2704	-5.1724
	6	-5.0815	-21.6107	-21.1884	-6.9240	60.3734	-5.0815
	7	-4.5611	4.0224	-20.2926	-6.8473	98.5157	-4.5611
	8	-4.4675	-16.5428	-24.0932	-7.2734	76.6828	-4.4675
	9	-4.4422	-14.6227	-27.8064	-7.0100	91.5883	-4.4422
	10	-4.2042	-23.7206	-20.6120	-7.1150	54.4230	-4.2042
**3** *h*TAAR1 EC_{50} = 330 nM	1	-6.1549	-36.0158	-22.0657	-7.4157	51.1603	-6.1549
	2	-5.3518	-29.8008	-19.8627	-7.2807	33.3252	-5.3518
	3	-5.1618	-32.7916	-21.6630	-7.6446	47.6420	-5.1618
	4	-5.0378	-22.3801	-22.9869	-7.3410	36.5296	-5.0378
	5	-4.8235	-29.6521	-26.7732	-9.1078	58.5681	-4.8235
	6	-4.7783	-36.1597	-18.8590	-7.1199	47.1015	-4.7783
	7	-4.7284	-15.7114	-24.0604	-7.4255	66.7364	-4.7284

	8	-4.5758	-41.5078	-21.9644	-7.1257	45.3505	-4.5758
	9	-4.3038	-32.8088	-16.6207	-7.3800	57.2294	-4.3038
	10	-4.0940	-23.5133	-23.4203	-7.7124	44.9587	-4.0940
**4** *h*TAAR1 EC_{50} = 2900 nM	1	-6.7972	-38.0066	-19.9762	-8.5578	36.1022	-6.7972
	2	-6.4669	-31.7055	-26.3440	-8.5643	35.6879	-6.4669
	3	-5.2996	-31.5329	-20.9002	-7.2604	27.5932	-5.2996
	4	-4.9246	-35.9998	-22.3115	-7.1174	29.8987	-4.9246
	5	-4.9028	-43.9267	-16.9371	-7.1407	31.2623	-4.9028
	6	-4.7972	-24.7701	-23.7803	-6.9858	42.8882	-4.7972
	7	-4.6714	-33.4334	-22.4209	-7.1023	26.2756	-4.6714
	8	-4.1795	-36.8714	-18.7170	-7.7451	40.7910	-4.1795
	9	-4.1160	-35.4978	-18.3147	-7.8329	35.8753	-4.1160
	10	-3.5924	-30.5785	-18.4338	-7.7216	55.8822	-3.5924
**5** *h*TAAR1 EC_{50} = 18 nM	1	-6.8864	-15.3874	-19.4253	-6.8157	61.5280	-6.8864
	2	-5.6539	-37.4924	-23.3899	-7.6575	57.3307	-5.6539
	3	-5.5774	-44.6408	-20.5300	-6.2518	41.3936	-5.5774
	4	-5.1564	-34.6700	-20.4675	-7.8286	52.0739	-5.1564
	5	-4.8274	-15.6303	-15.1902	-6.5498	81.3096	-4.8274
	6	-4.7919	-23.9413	-17.9953	-7.1429	93.6337	-4.7919
	7	-4.7877	-35.7884	-14.1168	-6.4866	61.8682	-4.7877
	8	-4.7084	-33.8470	-25.1072	-6.8446	69.2430	-4.7084
	9	-4.6625	-28.7702	-15.3947	-6.4696	83.7171	-4.6625
	10	-4.6447	-23.2218	-19.0323	-6.5708	86.0438	-4.6447
**6** *h*TAAR1 EC_{50} = 27 nM	1	-7.1321	-27.3425	-24.5538	-8.0463	36.1247	-7.1321
	2	-5.7943	-29.1738	-27.3264	-7.1039	47.8371	-5.7943
	3	-5.4822	-25.5393	-20.1710	-6.9600	58.5414	-5.4822
	4	-5.1047	-37.7120	-26.1677	-8.2777	51.7574	-5.1047
	5	-4.9999	-46.2657	-23.1260	-7.6310	33.5905	-4.9999
	6	-4.6681	-31.5881	-18.5909	-7.7499	77.4750	-4.6681
	7	-4.5985	-12.3695	-22.4607	-7.3528	83.3501	-4.5985
	8	-4.5097	-42.3900	-16.6551	-7.0548	69.8001	-4.5097
	9	-4.4846	-42.8587	-23.6359	-8.7270	44.0275	-4.4846
	10	-4.3983	-38.4380	-16.6926	-6.7957	75.4903	-4.3983
**7** *h*TAAR1 EC_{50} = 330 nM	1	-5.6156	-34.0375	-18.2656	-6.6363	49.0827	-5.6156
	2	-4.8597	-43.4411	-16.5363	-6.6186	41.2759	-4.8597
	3	-4.8347	-30.2570	-24.3336	-8.8323	52.7929	-4.8347
	4	-4.5006	-28.2558	-18.8814	-6.5490	54.8886	-4.5006
	5	-4.4203	-43.8437	-23.2039	-7.1151	48.7528	-4.4203
	6	-4.1130	-12.4839	-24.6910	-6.8143	67.1244	-4.1130
	7	-3.8998	-38.0914	-18.9045	-8.7773	51.6346	-3.8998
	8	-3.8162	-42.6710	-17.4310	-7.6884	68.7750	-3.8162
	9	-3.7170	-33.6538	-18.5100	-8.5352	50.8417	-3.7170
	10	-3.6921	-31.3123	-16.1915	-6.4398	56.4030	-3.6921

**8** *h*TAAR1 EC_{50} = 270 nM	1	-6.1963	-8.2608	-22.6254	-6.3889	72.0812	-6.1963
	2	-5.6427	-4.8025	-27.0414	-8.0195	69.5870	-5.6427
	3	-5.3609	-26.2730	-18.5638	-6.4766	58.1031	-5.3609
	4	-5.2596	-31.7425	-17.0212	-6.8020	46.5876	-5.2596
	5	-4.9436	-12.9168	-24.7847	-7.3462	79.2828	-4.9436
	6	-4.7103	-27.6818	-21.1480	-6.2127	64.9465	-4.7103
	7	-4.7087	-17.3333	-22.0571	-6.2409	81.4235	-4.7087
	8	-4.6418	-28.6830	-15.1207	-5.9025	85.2829	-4.6418
	9	-4.5947	-20.5807	-17.6996	-5.9701	91.7289	-4.5947
	10	-4.5442	1.9884	-23.1870	-6.9047	99.8646	-4.5442
**9** *h*TAAR1 EC_{50} = 580 nM	1	-4.9034	-8.0685	-19.1901	-7.5926	67.2207	-4.9034
	2	-4.6410	-34.1585	-23.0496	-6.8627	38.1620	-4.6410
	3	-4.5640	-27.7642	-23.3127	-7.1348	55.3547	-4.5640
	4	-3.9563	-33.3501	-19.7852	-6.7878	77.3748	-3.9563
	5	-3.8805	-37.2053	-21.6179	-6.9927	56.0357	-3.8805
	6	-3.8407	-19.9476	-24.6522	-7.7349	82.2431	-3.8407
	7	-3.8335	-38.4174	-18.8258	-7.4769	65.9573	-3.8335
	8	-3.6748	-24.8021	-15.4494	-8.7039	57.5789	-3.6748
	9	-3.4381	-32.3985	-22.5903	-8.2978	74.3877	-3.4381
	10	-3.2971	-18.9408	-24.7972	-7.2835	90.6948	-3.2971
**10** *h*TAAR1 EC_{50} = 27 nM	1	-6.5934	8.9311	-22.3847	-7.7689	99.7301	-6.5934
	2	-6.5830	-4.9196	-17.4415	-7.1127	88.1359	-6.5830
	3	-6.0577	-26.3055	-22.4049	-7.4483	59.6633	-6.0577
	4	-5.6404	-20.2312	-19.4029	-7.8313	81.9541	-5.6404
	5	-5.2880	14.4903	-14.7012	-6.5248	94.6400	-5.2880
	6	-4.8288	41.8128	-17.8033	-6.7447	140.9574	-4.8288
	7	-4.7683	-13.0329	-21.6526	-8.8171	78.7586	-4.7683
	8	-4.7230	-22.4103	-22.4719	-6.5913	65.3933	-4.7230
	9	-4.5278	2.1590	-18.2259	-6.7265	105.5904	-4.5278
	10	-4.4042	-15.1153	-26.5988	-8.9640	79.7274	-4.4042
**11** *h*TAAR1 EC_{50} = 29 nM	1	-6.5403	-19.5563	-14.5225	-5.6869	62.9233	-6.5403
	2	-5.9477	-20.4275	-20.2596	-7.1048	75.8684	-5.9477
	3	-5.5794	-3.8330	-20.3892	-6.4052	101.1149	-5.5794
	4	-4.9383	30.9561	-17.6864	-5.2506	151.1868	-4.9383
	5	-4.7740	-15.4236	-18.9327	-5.7316	94.4706	-4.7740
	6	-4.5015	-10.7896	-23.9205	-6.7852	128.0676	-4.5015
	7	-4.2028	3.8652	-24.0286	-6.0623	120.0662	-4.2028
	8	-4.0909	-10.6074	-20.7314	-5.3486	113.3602	-4.0909
	9	-3.9631	5.2321	-21.9646	-7.3569	138.6045	-3.9631
	10	-3.9504	34.7177	-25.9150	-5.8180	163.3991	-3.9504
**12** *h*TAAR1 EC_{50} = 59 nM	1	-6.1163	11.4990	-20.5053	-5.8631	69.3239	-6.1163
	2	-5.7853	11.5036	-18.0575	-5.7288	79.9161	-5.7853
	3	-5.3599	8.4386	-24.0783	-5.8641	83.9387	-5.3599

	4	-4.8889	2.5612	-15.9503	-5.7294	100.6678	-4.8889
	5	-4.8883	2.5565	-21.0558	-6.0650	100.6710	-4.8883
	6	-4.6423	-5.1033	-13.4893	-5.8212	74.4964	-4.6423
	7	-4.5529	-17.9619	-15.4083	-5.6704	78.6064	-4.5529
	8	-4.2597	3.7235	-25.8562	-6.7033	131.1304	-4.2597
	9	-4.0331	3.2943	-23.2267	-8.2936	108.5065	-4.0331
	10	-3.9450	0.8249	-24.8451	-6.9027	107.6811	-3.9450
**13** *h*TAAR1 EC_{50} = 140 nM	1	-5.1443	16.4722	-19.8323	-5.5490	105.4604	-5.1443
	2	-5.0409	3.9383	-16.3617	-5.4371	134.0432	-5.0409
	3	-4.8325	8.6100	-25.6461	-5.3101	98.0344	-4.8325
	4	-4.6731	39.6288	-20.7259	-5.7898	136.0357	-4.6731
	5	-4.6594	6.8118	-25.9618	-6.9038	103.8084	-4.6594
	6	-4.0228	26.3867	-18.9750	-5.2970	134.4154	-4.0228
	7	-3.8576	45.2923	-20.4072	-5.7987	173.8326	-3.8576
	8	-3.5531	24.4497	-29.6152	-7.3284	152.8035	-3.5531
	9	-3.4221	15.1629	-24.5529	-6.3183	129.3527	-3.4221
	10	-3.3680	21.4588	-20.2008	-5.4345	142.7564	-3.3680
**14** *h*TAAR1 EC_{50} = 230 nM	1	-6.3802	-8.4023	-24.5127	-9.3928	102.1423	-6.3802
	2	-6.3682	-10.9504	-19.2253	-6.3457	92.2616	-6.3682
	3	-5.8151	4.3051	-20.0694	-7.2129	78.1539	-5.8151
	4	-5.3961	-12.7965	-23.2141	-7.4878	70.0902	-5.3961
	5	-5.3954	-15.5105	-14.6270	-6.3440	75.9124	-5.3954
	6	-5.2506	-1.9586	-20.3966	-7.1428	70.1610	-5.2506
	7	-4.6664	1.0293	-19.9878	-6.2998	74.4637	-4.6664
	8	-4.6658	-6.6334	-22.8947	-6.0705	76.8432	-4.6658
	9	-4.5627	-9.0311	-25.9286	-6.0815	88.5458	-4.5627
	10	-4.3734	49.8935	-28.3964	-6.6913	142.9782	-4.3734
**15** *h*TAAR1 EC_{50} = 1540 nM	1	-4.6935	-38.7737	-23.0568	-7.3259	33.4581	-4.6935
	2	-4.6063	-23.3700	-21.6994	-7.4541	56.1294	-4.6063
	3	-4.6045	-33.9480	-21.5388	-6.8726	51.1592	-4.6045
	4	-4.5026	-26.2055	-21.1349	-7.3844	44.0496	-4.5026
	5	-4.1954	-27.8418	-17.1561	-7.6554	89.0193	-4.1954
	6	-4.1684	-27.6434	-17.1135	-7.9038	79.9943	-4.1684
	7	-3.7648	-21.1075	-17.9724	-8.5882	76.3813	-3.7648
	8	-3.6738	-12.7704	-22.6013	-6.8524	72.2670	-3.6738
	9	-3.6654	-34.7427	-22.1921	-6.9171	50.0567	-3.6654
	10	-3.6095	-33.4180	-24.0519	-7.8969	74.1526	-3.6095
**16** *h*TAAR1 EC_{50} = 730 nM	1	-7.2651	-17.4721	-25.5876	-8.9942	68.6147	-7.2651
	2	-5.3596	-21.4498	-24.2996	-5.9722	74.7530	-5.3596
	3	-4.9836	-30.8151	-21.6403	-8.1013	77.0429	-4.9836
	4	-4.9480	-20.4703	-26.1824	-6.0976	54.3206	-4.9480
	5	-4.8668	-17.2160	-23.9299	-6.7845	57.7022	-4.8668
	6	-4.5503	-22.8495	-17.8445	-7.0779	95.5715	-4.5503

	7	-4.5470	-10.6552	-24.8644	-6.5041	89.7699	-4.5470
	8	-4.1172	-7.0385	-26.3356	-6.5422	98.3558	-4.1172
	9	-4.0177	-9.9734	-22.9014	-6.0367	95.1175	-4.0177
	10	-3.9598	-16.3538	-17.9432	-7.2324	89.4466	-3.9598
**17** *h*TAAR1 EC_{50} = 2260 nM	1	-5.9924	-19.6231	-26.9717	-8.0984	64.6049	-5.9924
	2	-5.9542	-17.7467	-24.1101	-5.7430	87.8876	-5.9542
	3	-5.4397	-17.1603	-19.2931	-5.8601	94.9349	-5.4397
	4	-5.0130	-7.8555	-16.3583	-5.5924	110.2700	-5.0130
	5	-4.9463	-11.7779	-20.7162	-6.5320	127.3136	-4.9463
	6	-4.8433	-21.3077	-23.4846	-6.9001	84.2078	-4.8433
	7	-4.2519	-6.6178	-19.4581	-5.6316	90.9768	-4.2519
	8	-4.2384	-18.1158	-23.0880	-7.1305	122.9625	-4.2384
	9	-4.2357	-10.2552	-27.7198	-8.0031	133.2605	-4.2357
	10	-4.2276	19.8144	-25.6749	-6.3408	105.5253	-4.2276
**18** *h*TAAR1 EC_{50} = 18 nM	1	-5.8995	-19.4477	-21.5990	-6.4736	66.7397	-5.8995
	2	-5.8858	-8.7701	-23.5652	-7.2797	97.4173	-5.8858
	3	-5.8241	84.7990	-17.0127	-5.4463	85.9081	-5.8241
	4	-5.3851	-4.7572	-22.4809	-5.8050	110.2812	-5.3851
	5	-5.2534	12.9457	-17.8497	-5.3066	112.5593	-5.2534
	6	-5.1653	-10.6398	-19.6421	-5.4023	83.8682	-5.1653
	7	-5.1115	-15.4849	-23.9871	-5.7397	83.0481	-5.1115
	8	-4.9875	-20.1539	-20.4906	-5.8840	78.1003	-4.9875
	9	-4.9202	15.9377	-26.1015	-6.0793	130.2157	-4.9202
	10	-4.4274	-15.7983	-22.3029	-6.1400	98.4870	-4.4274
**19** *h*TAAR1 EC_{50} = 27 nM	1	-5.8673	-29.4537	-22.2913	-6.3345	66.8797	-5.8673
	2	-5.1821	-4.7192	-16.6009	-6.0338	85.4327	-5.1821
	3	-5.1051	-29.3070	-21.2214	-7.6320	54.3333	-5.1051
	4	-5.0356	-31.7821	-19.8464	-6.2416	56.2526	-5.0356
	5	-5.0229	2.7465	-20.9697	-6.2478	96.8197	-5.0229
	6	-5.0225	-34.7393	-20.7167	-7.6373	68.4762	-5.0225
	7	-5.0009	-30.7104	-24.0461	-7.9744	67.5973	-5.0009
	8	-4.9965	-4.4260	-18.8881	-6.1356	90.9592	-4.9965
	9	-4.9305	-32.7494	-21.6785	-7.1063	68.4227	-4.9305
	10	-4.7565	-31.6748	-20.7138	-6.3672	57.5943	-4.7565
**20** *h*TAAR1 EC_{50} = 360 nM	1	-5.1471	-5.4932	-23.5130	-8.2849	58.9700	-5.1471
	2	-4.8847	-15.9983	-24.5901	-6.9573	51.1508	-4.8847
	3	-4.6718	-20.5389	-18.2102	-7.2898	55.0088	-4.6718
	4	-4.3880	-18.3302	-22.5202	-8.1003	72.3603	-4.3880
	5	-4.3529	-26.6350	-19.8866	-7.8173	43.5059	-4.3529
	6	-4.0570	-19.9268	-18.8019	-6.8934	57.2523	-4.0570
	7	-3.9721	-22.0313	-15.2656	-7.5710	85.4728	-3.9721
	8	-3.8822	-19.4499	-18.7427	-7.3388	55.6253	-3.8822
	9	-3.7071	-11.9078	-23.0672	-8.0938	64.0509	-3.7071

	10	-3.5732	-14.0306	-23.8937	-7.3378	89.3986	-3.5732
**21** *h*TAAR1 EC_{50} = 9 nM	1	-7.1321	-27.3425	-24.5538	-8.0463	36.1247	-7.1321
	2	-5.7943	-29.1738	-27.3264	-7.1039	47.8371	-5.7943
	3	-5.4822	-25.5393	-20.1710	-6.9600	58.5414	-5.4822
	4	-5.1047	-37.7120	-26.1677	-8.2777	51.7574	-5.1047
	5	-4.9999	-46.2657	-23.1260	-7.6310	33.5905	-4.9999
	6	-4.6681	-31.5881	-18.5909	-7.7499	77.4750	-4.6681
	7	-4.5985	-12.3695	-22.4607	-7.3528	83.3501	-4.5985
	8	-4.5097	-42.3900	-16.6551	-7.0548	69.8001	-4.5097
	9	-4.4846	-42.8587	-23.6359	-8.7270	44.0275	-4.4846
	10	-4.3983	-38.4380	-16.6926	-6.7957	75.4903	-4.3983
**22** *h*TAAR1 EC_{50} = 67 nM	1	-6.2967	-40.9669	-17.0249	-8.0597	26.8154	-6.2967
	2	-5.2174	-37.8697	-26.1858	-9.4604	25.5665	-5.2174
	3	-4.5791	-37.6446	-20.3492	-7.1420	30.0341	-4.5791
	4	-4.4556	-43.9240	-23.7566	-7.0644	19.1589	-4.4556
	5	-4.0838	-41.3367	-19.3330	-7.0497	23.2138	-4.0838
	6	-3.7624	-39.7435	-21.5506	-7.1900	38.5874	-3.7624
	7	-3.5559	-32.3070	-17.7689	-7.4242	48.9301	-3.5559
	8	-3.3917	-42.3285	-21.6941	-7.2785	82.8841	-3.3917
	9	-3.0253	-26.8048	-19.8943	-7.2886	77.0915	-3.0253
	10	-2.8186	-33.4570	-21.0733	-7.1998	37.6987	-2.8186
**23** *h*TAAR1 EC_{50} = 23 nM	1	-6.6376	-35.2316	-25.3815	-7.1150	38.1100	-6.6376
	2	-5.9792	-32.8052	-21.4647	-7.0396	41.2152	-5.9792
	3	-5.7333	-25.3283	-24.7910	-7.6761	42.7956	-5.7333
	4	-5.3401	-24.8614	-21.8836	-7.7691	34.6886	-5.3401
	5	-4.8696	-41.0606	-20.3802	-7.3974	28.5993	-4.8696
	6	-4.7172	-32.2484	-20.8973	-7.3405	34.3990	-4.7172
	7	-4.6542	-33.3414	-18.8737	-7.2548	42.7109	-4.6542
	8	-4.6188	-35.3914	-23.0510	-6.9705	32.7379	-4.6188
	9	-4.4178	-27.7386	-18.7359	-7.4912	43.9964	-4.4178
	10	-4.1274	-34.0951	-23.7766	-7.1517	54.4393	-4.1274
**24** *h*TAAR1 EC_{50} = 21 nM	1	-6.7660	-39.2142	-21.2077	-7.7919	35.2301	-6.7660
	2	-6.3869	-26.7505	-23.6495	-8.5964	36.1577	-6.3869
	3	-5.8919	-40.2858	-25.7987	-8.3650	29.6380	-5.8919
	4	-5.3564	-18.0542	-23.1650	-7.4576	39.9734	-5.3564
	5	-4.8150	-35.0663	-22.8922	-7.3870	64.5351	-4.8150
	6	-4.7168	-31.2658	-19.9409	-8.1314	46.3817	-4.7168
	7	-4.4596	-37.7640	-22.7318	-8.3238	42.2492	-4.4596
	8	-4.4380	-43.1819	-22.8206	-7.5171	32.1591	-4.4380
	9	-4.4122	-35.2245	-22.3556	-7.5842	39.8276	-4.4122
	10	-4.0434	-35.2589	-15.1747	-7.9072	48.1488	-4.0434
**25** *h*TAAR1 EC_{50} = 143 nM	1	-6.0587	-40.0817	-18.9627	-8.2905	40.0792	-6.0587
	2	-5.7859	-37.4548	-22.4010	-7.3645	40.7768	-5.7859

	3	-4.8708	-33.1073	-22.3470	-7.6797	38.5799	-4.8708
	4	-4.3526	-35.7069	-24.4994	-8.1462	44.8309	-4.3526
	5	-4.2661	-28.6965	-19.5447	-7.7417	63.8305	-4.2661
	6	-4.2294	-40.7538	-22.0262	-7.6565	43.6701	-4.2294
	7	-3.6653	-41.9682	-22.6719	-7.3594	86.0401	-3.6653
	8	-3.0846	-38.6781	-19.8189	-7.5563	48.8080	-3.0846
	9	-2.8487	-7.0492	-25.0847	-7.8930	106.0186	-2.8487
	10	-2.2486	-26.3095	-18.7892	-7.8561	78.0564	-2.2486
**26** *h*TAAR1 EC_{50} = 31 nM	1	-6.5172	-19.2759	-23.3346	-8.6370	63.0470	-6.5172
	2	-6.4759	-26.3456	-15.8418	-7.6644	51.4135	-6.4759
	3	-6.2835	-28.1116	-24.4181	-9.1002	48.5919	-6.2835
	4	-5.2983	-25.9763	-24.3488	-7.6943	69.6108	-5.2983
	5	-5.1907	-28.9903	-21.4981	-7.3354	40.4314	-5.1907
	6	-4.9580	-30.8108	-21.5106	-8.2095	35.7579	-4.9580
	7	-4.8735	-28.9794	-20.5045	-8.0553	55.5786	-4.8735
	8	-4.6469	-26.3943	-22.1587	-8.5537	61.7573	-4.6469
	9	-4.5080	-24.8827	-23.8021	-7.6090	41.9113	-4.5080
	10	-4.4422	-20.7882	-21.8810	-8.0998	70.0162	-4.4422
**27** *h*TAAR1 EC_{50} = 150 nM	1	-5.9241	-39.8122	-16.4641	-8.2010	48.7249	-5.9241
	2	-5.6790	-35.9067	-22.3257	-7.7803	51.8193	-5.6790
	3	-4.9674	-32.0498	-21.5616	-7.9531	42.0947	-4.9674
	4	-4.2378	-40.1425	-22.0259	-7.8717	48.0580	-4.2378
	5	-4.1524	-27.9701	-19.3901	-7.8495	73.3024	-4.1524
	6	-3.5650	-41.2960	-22.3936	-7.5027	90.2077	-3.5650
	7	-3.1667	-37.6019	-18.9126	-7.5024	54.1272	-3.1667
	8	-2.5406	15.6275	-24.8262	-7.8442	124.6538	-2.5406
	9	-2.4258	15.6385	-22.8453	-7.7564	121.6276	-2.4258
	10	-1.1355	15.9452	-21.8342	-7.4511	119.5627	-1.1355
**28** *h*TAAR1 EC_{50} = 10,000 nM	1	-5.0876	8.5288	-31.0970	-8.2167	69.7980	-5.0876
	2	-4.8677	-14.8992	-24.1368	-7.2600	90.2528	-4.8677
	3	-4.8613	-2.1946	-24.1086	-7.3924	100.3444	-4.8613
	4	-4.8151	-12.4050	-22.9402	-7.5325	80.3298	-4.8151
	5	-4.0645	3.7944	-25.5766	-7.2105	107.4177	-4.0645
	6	-3.3866	-7.9762	-32.1872	-7.3126	124.0533	-3.3866
	7	-3.3620	6.9004	-34.7742	-8.9816	145.1023	-3.3620
	8	-3.2907	17.1154	-34.9518	-8.6153	135.9447	-3.2907
	9	-2.9559	0.4934	-26.3173	-8.5449	138.4343	-2.9559
	10	-2.5781	-9.2297	-23.2777	-8.7709	132.2009	-2.5781
**29** *h*TAAR1 EC_{50} = 165 nM	1	-5.5681	-2.7817	-21.7057	-6.7897	98.0218	-5.5681
	2	-5.4285	6.4681	-18.3928	-8.4757	90.3379	-5.4285
	3	-5.0490	-22.5101	-24.2387	-7.9250	77.6759	-5.0490
	4	-4.8980	5.7462	-20.2321	-7.2826	68.6273	-4.8980
	5	-4.7759	-7.2525	-22.2122	-6.7125	74.0144	-4.7759

	6	-4.7746	-7.0220	-24.6720	-7.0475	86.0462	-4.7746
	7	-4.7402	-8.7031	-24.4684	-7.8356	103.4484	-4.7402
	8	-4.5584	-9.0113	-22.3071	-8.1303	78.4164	-4.5584
	9	-4.3533	-9.1699	-21.9990	-6.6961	62.8561	-4.3533
	10	-4.1758	-12.5683	-21.5935	-7.0961	55.2545	-4.1758
**30** *h*TAAR1 EC_{50} = 41 nM	1	-5.7542	12.9506	-24.9927	-7.1019	99.4690	-5.7542
	2	-5.6246	-6.8423	-19.7980	-6.8472	101.4978	-5.6246
	3	-5.1191	12.0748	-23.5430	-7.2725	94.8400	-5.1191
	4	-4.9574	5.4876	-22.0978	-7.4241	95.0573	-4.9574
	5	-4.9034	37.4219	-26.6170	-7.4857	93.0567	-4.9034
	6	-4.6665	-4.3461	-24.9162	-7.8693	105.3818	-4.6665
	7	-4.3741	26.1608	-20.9977	-5.9105	136.8058	-4.3741
	8	-4.3737	1.3348	-17.3248	-5.7469	75.6871	-4.3737
	9	-4.1238	-2.6108	-27.7652	-7.2259	119.6468	-4.1238
	10	-4.0725	-4.1149	-21.9351	-6.2197	115.9044	-4.0725
**31** *h*TAAR1 EC_{50} = 2670 nM	1	-5.2125	-12.8271	-24.0601	-7.4513	82.4449	-5.2125
	2	-5.1545	-24.6554	-23.4979	-7.1135	64.2694	-5.1545
	3	-4.9737	8.7000	-26.0391	-7.5866	68.6277	-4.9737
	4	-4.7171	-1.2377	-20.8628	-8.3721	72.5991	-4.7171
	5	-4.5298	2.3445	-19.5213	-9.7540	112.1964	-4.5298
	6	-4.4678	-4.6109	-19.0481	-7.5815	55.9544	-4.4678
	7	-4.4568	-13.3755	-26.6144	-8.2111	76.9502	-4.4568
	8	-4.3886	-8.1993	-23.0638	-8.0742	49.1586	-4.3886
	9	-4.2369	2.2722	-21.5274	-8.4353	109.6801	-4.2369
	10	-4.0950	-17.4087	-23.7741	-8.2173	64.1929	-4.0950
**32** *h*TAAR1 EC_{50} = 490 nM	1	-5.6820	-27.7089	-27.7691	-9.4364	59.2260	-5.6820
	2	-5.6674	-15.1882	-25.9686	-8.8388	54.6854	-5.6674
	3	-5.5078	-29.6561	-23.4904	-7.8403	46.0487	-5.5078
	4	-5.3184	-29.7925	-27.0327	-8.7333	43.9000	-5.3184
	5	-5.0350	-12.1507	-25.3950	-7.6953	56.3332	-5.0350
	6	-4.6034	-31.2222	-23.6655	-8.3929	51.1106	-4.6034
	7	-4.2767	-11.5550	-21.1087	-8.3793	71.0925	-4.2767
	8	-4.2250	-21.5578	-27.1114	-9.1363	57.3157	-4.2250
	9	-4.1119	-14.3344	-22.3300	-7.8000	76.4074	-4.1119
	10	-3.8845	-14.2602	-22.8312	-7.7458	82.7195	-3.8845
**33** *h*TAAR1 EC_{50} = 67 nM	1	-6.7540	-27.4888	-21.3524	-7.5644	34.9690	-6.7540
	2	-5.7917	-30.7316	-23.7091	-7.4657	37.8438	-5.7917
	3	-5.5037	-20.0455	-26.4736	-8.0018	38.7906	-5.5037
	4	-5.1330	-22.6209	-21.6061	-7.9489	50.6076	-5.1330
	5	-4.7100	-30.0040	-23.2812	-7.5560	32.0300	-4.7100
	6	-4.5396	-32.2064	-24.2715	-7.7641	38.3439	-4.5396
	7	-4.4099	-37.2445	-23.6388	-7.9031	30.5661	-4.4099
	8	-4.0668	-24.3428	-25.9083	-8.8428	39.3740	-4.0668

	9	-3.8794	-15.9126	-23.5651	-7.7942	49.9351	-3.8794
	10	-3.8687	-16.4945	-23.6610	-7.5623	45.7328	-3.8687
**34** *h*TAAR1 EC_{50} = 11 nM	1	-6.9372	-42.4904	-20.9899	-7.4075	26.5656	-6.9372
	2	-6.3771	-42.3133	-19.9400	-7.5713	26.9569	-6.3771
	3	-5.7721	-40.2232	-25.1115	-7.8061	29.3558	-5.7721
	4	-5.6896	-40.3991	-24.8415	-7.9584	24.7990	-5.6896
	5	-4.9783	-40.1120	-22.6367	-7.6169	29.0833	-4.9783
	6	-4.9385	-45.3749	-24.2207	-8.6896	20.3436	-4.9385
	7	-4.5878	-45.6488	-18.4336	-7.3962	22.2142	-4.5878
	8	-4.5734	-43.7929	-23.8062	-7.6180	22.5100	-4.5734
	9	-4.5461	-38.8536	-18.3512	-8.6099	29.6434	-4.5461
	10	-4.2763	-40.6445	-22.3187	-7.6997	32.1229	-4.2763
**35** *h*TAAR1 EC_{50} = 26 nM	1	-6.4499	-19.9664	-23.1239	-8.0984	84.5830	-6.4499
	2	-5.6395	45.5510	-22.6900	-8.4316	80.4564	-5.6395
	3	-5.4829	-13.9273	-29.1873	-8.8587	92.2526	-5.4829
	4	-5.3185	-18.7812	-22.0211	-7.5539	54.1103	-5.3185
	5	-4.7950	-16.8873	-24.8529	-7.1051	86.4337	-4.7950
	6	-4.5993	-0.7258	-24.2479	-6.9584	101.3593	-4.5993
	7	-4.5207	-13.3521	-19.3669	-8.3583	71.0713	-4.5207
	8	-4.3765	-16.9550	-25.2527	-7.0470	73.6032	-4.3765
	9	-4.3654	-23.7325	-24.1790	-7.0855	83.7357	-4.3654
	10	-4.3178	-9.1482	-25.3034	-7.1636	83.2448	-4.3178
**36** *h*TAAR1 EC_{50} = 12 nM	1	-6.0151	-0.1375	-24.5698	-7.5687	97.5924	-6.0151
	2	-5.1646	-8.6264	-23.0521	-8.2809	104.9822	-5.1646
	3	-4.4914	-6.4538	-30.7809	-9.6461	74.2706	-4.4914
	4	-4.2140	1.1782	-20.9086	-8.0394	106.2322	-4.2140
	5	-4.1202	18.0031	-23.3348	-7.9856	111.5072	-4.1202
	6	-4.1108	6.2332	-26.6407	-8.5902	109.8328	-4.1108
	7	-3.9793	12.0944	-26.8630	-7.9858	108.2645	-3.9793
	8	-3.8812	25.4461	-26.9941	-8.4958	112.3095	-3.8812
	9	-3.8181	-14.7079	-24.8460	-8.4565	87.9899	-3.8181
	10	-3.6731	-4.5015	-17.6121	-8.3705	87.5786	-3.6731
**37** *h*TAAR1 EC_{50} = 17 nM	1	-6.6828	-26.8331	-20.8239	-7.5809	35.8364	-6.6828
	2	-6.0009	-21.6556	-26.8579	-8.7243	37.9428	-6.0009
	3	-5.9975	-28.7963	-20.2330	-7.9749	39.5236	-5.9975
	4	-5.4550	-25.0046	-25.4479	-7.7344	41.5010	-5.4550
	5	-5.0607	-18.1754	-25.5084	-8.6690	64.1625	-5.0607
	6	-4.7328	-31.5658	-22.3384	-7.5040	39.3946	-4.7328
	7	-4.5933	-33.0160	-25.5540	-9.7079	38.6451	-4.5933
	8	-4.5601	-25.9295	-19.2421	-7.4896	38.5164	-4.5601
	9	-4.4189	-25.5238	-20.7732	-7.7145	40.2514	-4.4189
	10	-4.3026	-23.3862	-16.6883	-7.6914	47.3670	-4.3026

Table S4. The calculated scoring function Glob-Prod values based on the MIFs H, N1, DRY, O are reported (compounds **1a-10a** as candidates, being **S18616** the template). The most promising **1a-3a** are depicted in pink.

Candidate	Glob-Prod	H	N1	DRY	O
**1a**	0.3499	0.5475	0.2631	0.2196	0.4741
**3a**	0.3167	0.5138	0.1492	0.2080	0.1722
**2a**	0.3154	0.5391	0.3046	0.2451	0.2861
**6a**	0.2962	0.5887	0.1752	0.2046	0.0100
**4a**	0.2841	0.4656	0.1460	0.2228	0.1849
**5a**	0.2838	0.5084	0.1717	0.2491	0.3139
**9a**	0.2692	0.5431	0.1675	0.2268	0.0100
**10a**	0.2463	0.5473	0.1285	0.2361	0.0764
**8a**	0.2435	0.5948	0.2953	0.2333	0.0100
**7a**	0.1051	0.5535	0.2190	0.2266	0.1224

Table S5. The calculated scoring function Glob-Prod values based on the MIFs H, N1, DRY, O are reported (compounds **2-37** as candidates, being **S18616** the template). The benzyl-based analogues of **S18616** (**2**-**4**; *h*TAAR1 EC_{50} = 154-2,900 nM) and the phenyl (hetero)alkyl-containing compounds (**5**-**21**; *h*TAAR1 EC_{50} = 9-2,260 nM) are highlighted in cyan and orange, respectively. The hetero-containing ones are showed in italic. The **S18616** phenyl-based- analogues (**22**-**37**; *h*TAAR1 EC_{50} = 11-10,000 nM) are reported in white.

Candidate	*h*TAAR1 EC_{50} (nM)	Glob-Prod	H	H1	DRY	O
**2**	154	0.6494	0.7427	0.4930	0.4718	0.5751
**4**	2900	0.5988	0.6653	0.5636	0.3655	0.6997
**3**	330	0.5961	0.7004	0.3643	0.3402	0.4351
**19**	27	0.5047	0.6382	0.5238	0.3318	0.4245
**18**	18	0.4735	0.6479	0.4457	0.2844	0.2094
**7**	330	0.4472	0.5941	0.4583	0.3324	0.2771
**6**	27	0.4429	0.6454	0.3705	0.3268	0.4258
20	*360*	0.4284	0.6326	0.3349	0.3769	0.2624
9	*580*	*0.4250*	*0.5820*	*0.3961*	*0.3211*	*0.2954*
21	*9*	*0.4233*	*0.6057*	*0.3220*	*0.3011*	*0.4361*
**17**	2260	0.4226	0.5576	0.3092	0.3042	0.3115
13	*140*	*0.4199*	*0.5991*	*0.4146*	*0.2699*	*0.5362*
14	*230*	*0.4170*	*0.6174*	*0.3152*	*0.3276*	*0.3228*
**15**	1540	0.4090	0.6071	0.2481	0.3155	0.1255
**30**	41	0.4076	0.5788	0.4256	0.3334	0.5153
**24**	21	0.4069	0.5812	0.4457	0.4559	0.1573
**29**	165	0.4059	0.6185	0.3473	0.3105	0.5780
**33**	67	0.4013	0.6359	0.2023	0.3530	0.1605
**23**	23	0.3951	0.6372	0.1654	0.3468	0.1696
**26**	31	0.3920	0.6108	0.3636	0.3341	0.1622
8	*270*	*0.3911*	*0.5898*	*0.3199*	*0.2946*	*0.2012*
**16**	730	0.3906	0.5842	0.3621	0.3016	0.1646
**25**	143	0.3762	0.6005	0.3345	0.4022	0.0919
**35**	26	0.3712	0.5832	0.3206	0.3409	0.1339

**22**	67	0.3651	0.5737	0.1765	0.3234	0.1345
**36**	12	0.3633	0.5520	0.2477	0.3140	0.2209
**5**	18	0.3606	0.6895	0.3511	0.2926	0.1541
**34**	11	0.3555	0.5716	0.2967	0.3617	0.1771
**27**	150	0.3551	0.5921	0.3294	0.3949	0.0924
**10**	27	0.3537	0.5998	0.2357	0.2957	0.1764
**32**	490	0.3533	0.6274	0.3133	0.2603	0.1591
**28**	10000	0.3359	0.5787	0.1788	0.3712	0.0853
**11**	29	0.3164	0.6058	0.1990	0.2526	0.1273
**12**	59	0.3144	0.6285	0.2903	0.2625	0.1762
**31**	2670	0.3079	0.5137	0.2600	0.2636	0.0680
**37**	17	0.3051	0.5580	0.2278	0.3221	0.1311

Table S6. Ten top scored docking positioning of **1a-10a** [59] at the *h*TAAR1 (MOE software). The predicted ΔG value of each protein-ligand complex has been reported, as calculated in terms of final scoring function (S, as Kcal/mol). The top-scored pose for each compound is highlighted in cyan.

Compound	Pose	S	E_conf	E_place	E_score1	E_refine	E_score2
**1a**	1	-3.1695	39.7850	-23.4114	-7.8680	208.9993	-3.1695
	2	-2.5717	24.3562	-18.0866	-6.0924	191.1877	-2.5717
	3	-2.2160	39.7097	-18.5221	-5.4059	195.7730	-2.2160
	4	-1.8085	14.7057	-18.2489	-7.1554	182.9133	-1.8085
	5	-1.5860	38.1182	-15.5368	-6.1179	170.6386	-1.5860
	6	-1.5235	15.9138	-13.5874	-5.4332	186.0287	-1.5235
	7	-1.3555	30.3358	-15.9548	-6.1640	225.3294	-1.3555
	8	-1.3196	66.2664	-13.0831	-7.1754	180.1840	-1.3196
	9	-0.9655	33.0560	-16.9291	-6.5175	163.5597	-0.9655
	10	-0.9648	39.8692	-6.4055	-5.4730	173.4964	-0.9648
**2a**	1	-3.1254	1.7335	-22.5884	-7.0201	185.3491	-3.1254
	2	-2.3232	8.9617	-17.0826	-4.9698	190.2928	-2.3232
	3	-2.2945	17.7624	-24.9155	-6.6380	135.1178	-2.2945
	4	-1.6866	26.1267	-13.8330	-4.9297	207.7895	-1.6866
	5	-1.6354	11.9378	-15.8383	-5.1573	176.1240	-1.6354
	6	-1.4561	14.3683	-16.2898	-6.2027	166.6171	-1.4561
	7	-1.1047	7.6837	-20.4426	-6.2755	198.7422	-1.1047
	8	-0.8684	9.1266	-12.2015	-5.0801	183.0762	-0.8684
	9	-0.8217	61.6527	-16.5854	-5.0754	210.5317	-0.8217
	10	-0.5417	23.0917	-16.3569	-6.0353	235.8113	-0.5417
**3a**	1	-3.1408	3.0408	-23.5180	-5.5747	159.0475	-3.1408
	2	-2.8184	3.6954	-15.0186	-5.5231	196.4089	-2.8184
	3	-1.8132	-0.0241	-23.6092	-5.5833	168.2438	-1.8132
	4	-1.8085	91.7966	-11.5544	-5.9033	242.2801	-1.8085
	5	-0.7493	30.1222	-19.5386	-5.7995	258.9114	-0.7493
	6	0.2122	20.4458	-16.3604	-5.7466	210.7669	0.2122
	7	0.3028	111.1427	-16.3514	-6.3853	269.6474	0.3028
	8	1.1642	21.1243	-15.8258	-6.2218	235.2227	1.1642

	9	1.2276	53.3243	-19.3461	-5.6746	300.0271	1.2276
	10	2.0934	41.8743	-19.3917	-5.2961	274.0556	2.0934
**4a**	1	-2.6083	11.1736	-10.6675	-4.5770	175.2429	-2.6083
	2	-1.9117	4.0776	-17.8238	-4.1364	194.3487	-1.9117
	3	-1.5523	76.9709	-18.9652	-4.7434	247.0259	-1.5523
	4	-1.0867	13.1575	-7.1110	-4.2048	194.2562	-1.0867
	5	-1.0050	35.0532	-17.8631	-6.8073	226.3362	-1.0050
	6	-0.8958	9.7662	-18.3871	-3.8440	221.7271	-0.8958
	7	-0.4933	162.6557	-13.9580	-5.0616	252.7883	-0.4933
	8	-0.3985	-2.2733	-15.7303	-3.7705	251.6706	-0.3985
	9	0.1997	11.7054	-22.5034	-5.4538	318.5023	0.1997
	10	0.9285	74.0496	-11.9815	-4.7096	328.8462	0.9285
**5a**	1	-3.1141	13.7655	-15.8626	-4.1770	184.3825	-3.1141
	2	-2.5561	22.9830	-18.5702	-4.0677	161.3013	-2.5561
	3	-1.7673	72.7071	-14.2557	-3.8524	182.4056	-1.7673
	4	-1.6489	99.9865	-10.2883	-3.3533	188.5953	-1.6489
	5	-1.4228	116.8099	-13.0224	-3.1562	180.1016	-1.4228
	6	-1.3112	60.0023	-16.9652	-3.9120	214.2038	-1.3112
	7	-0.9906	79.3211	-11.2642	-3.1254	189.9630	-0.9906
	8	-0.9636	53.6107	-17.1162	-4.8920	196.1929	-0.9636
	9	0.2416	32.5744	-11.4511	-3.3555	273.8983	0.2416
	10	0.5239	69.7157	-12.3461	-3.1219	200.1670	0.5239
**6a**	1	-2.9174	81.3393	-12.7204	-1.2391	319.5895	-2.9174
	2	0.8933	132.0956	-9.9858	-1.3394	322.7433	0.8933
	3	1.7270	140.6551	-11.4462	-1.6347	390.6113	1.7270
	4	3.0155	69.6927	-7.0988	-2.4115	486.9999	3.0155
	5	3.2813	106.9135	-10.3179	-4.1189	340.8881	3.2813
	6	3.5249	100.0661	-8.2190	-1.6014	456.3620	3.5249
	7	4.0113	123.6151	-16.5476	-3.8286	391.3462	4.0113
	8	4.8094	88.9776	-11.8288	-5.3447	573.6596	4.8094
	9	4.8850	72.3250	-12.9699	-1.4972	343.3426	4.8850
	10	5.3481	40.2198	-17.1820	-3.7854	375.1483	5.3481
**7a**	1	3.9286	255.6012	-8.3833	-3.6899	441.4577	3.9286
	2	4.6972	290.5201	-23.2742	-2.8002	541.5862	4.6972
	3	4.8943	213.9906	-14.5667	-3.0993	457.7971	4.8943
	4	5.1335	201.9324	-8.3003	-1.4715	376.7648	5.1335
	5	6.0859	223.4280	-13.4008	-1.1211	435.0092	6.0859
	6	7.2675	187.8137	-13.9482	-2.1325	456.8268	7.2675
	7	7.9419	158.4915	-13.0340	-2.6253	463.5701	7.9419
	8	8.0137	203.9614	-17.2135	-1.8021	504.2595	8.0137
	9	8.0624	256.6322	-17.4123	-3.4894	514.2152	8.0624
	10	8.2770	166.7484	-16.6370	-4.4629	471.9864	8.2770
**8a**	1	0.4922	223.0022	-8.8665	-0.7471	359.6047	0.4922
	2	0.8809	133.1885	-21.5195	-1.5544	402.7855	0.8809
	3	2.2743	147.8503	-17.1259	-0.9820	360.9749	2.2743
	4	3.2984	107.1270	-5.1549	-1.2044	431.6612	3.2984

	5	3.6061	102.1159	-13.4230	-0.9220	424.0291	3.6061
	6	3.9386	196.0728	-13.5427	-0.7073	429.5690	3.9386
	7	4.7329	195.8803	-13.9889	-2.8246	440.7139	4.7329
	8	4.7864	64.6180	-7.2837	0.0358	352.5703	4.7864
	9	5.3541	144.1443	-13.2699	-0.0041	473.2814	5.3541
	10	5.7769	248.6318	-4.4040	-2.1394	423.1394	5.7769
**9a**	1	-1.4568	46.5824	-16.8081	-2.6719	268.8596	-1.4568
	2	0.0996	129.3075	-13.4415	-1.9082	301.5902	0.0996
	3	0.7424	18.1360	-18.0956	-0.6348	283.8612	0.7424
	4	2.8943	44.3939	-9.6439	-1.3603	325.5799	2.8943
	5	3.7381	53.4073	-10.2945	-1.0197	346.0813	3.7381
	6	3.8308	32.9870	-8.6431	-1.4346	351.2408	3.8308
	7	4.2320	154.8272	-12.2616	-1.9483	358.0657	4.2320
	8	4.5264	9.2886	-3.2174	-1.3169	349.7622	4.5264
	9	4.9422	55.8297	-14.0949	-0.9339	425.2249	4.9422
	10	5.0738	176.9914	2.0916	-0.6523	415.1421	5.0738
**10a**	1	0.4922	223.0022	-8.8665	-0.7471	359.6047	0.4922
	2	0.8809	133.1885	-21.5195	-1.5544	402.7855	0.8809
	3	2.2743	147.8503	-17.1259	-0.9820	360.9749	2.2743
	4	3.2984	107.1270	-5.1549	-1.2044	431.6612	3.2984
	5	3.6061	102.1159	-13.4230	-0.9220	424.0291	3.6061
	6	3.9386	196.0728	-13.5427	-0.7073	429.5690	3.9386
	7	4.7329	195.8803	-13.9889	-2.8246	440.7139	4.7329
	8	4.7864	64.6180	-7.2837	0.0358	352.5703	4.7864
	9	5.3541	144.1443	-13.2699	-0.0041	473.2814	5.3541
	10	5.7769	248.6318	-4.4040	-2.1394	423.1394	5.7769

Table S7. Calculated descriptors related to toxicity properties, cytochrome inhibition and lethal dose via mouse oral administration have been explored, as well as Pan Assay Interference structures (PAINS) analysis. The analysis has been reported for **1a**-**6a** [59] and for the reference **S18616** [59]. Reliability index values for a number of descriptors are shown as R.I. (values higher than 0.30 are ranked as reliable by the software).

**Comp.**	**CYP2C9**[a] Inhibitor (IC_{50} < 10mM) (R.I. ≥ 0.30)	**CYP3A4** [a] Inhibitor (IC_{50} < 10mM) (R.I. ≥ 0.30)	**LD_{50} (mg/kg)**[b] Mouse oral (R.I. ≥ 0.30)	**PAINS DA SWISSADME**
**1a**	0.01	0.00	390	0 ALERT
**2a**	0.01	0.00	400	0 ALERT
**3a**	0.02	0.27	390	0 ALERT
**4a**	0.00	0.00	280	0 ALERT
**5a**	0.00	0.00	280	0 ALERT
**6a**	0.06	0.04	370	0 ALERT
**S18616**	0.02	0.02	67	0 ALERT

a Prediction of the ligand inhibitor behavior towards cytochrome CYP2C9 and CYP3A4; b Acute toxicity (LD_{50}) for mouse after oral administration.
